# Scaling up evidence-based interventions to reduce maternal and child undernutrition in 125 countries: a cost-benefit analysis

**DOI:** 10.1186/s12939-026-02872-5

**Published:** 2026-05-12

**Authors:** Nick Scott, Tharindu Wickramaarachchi, Natalia Rovelo-Velázquez, Mireya Vilar-Compte

**Affiliations:** 1 Disease Elimination Program, Burnet Institute, Melbourne, Australia; 2 School of Public Health and Preventive Medicine, Monash University, Melbourne, Australia; 3 Health, Nutrition & Population Global Practice, World Bank Group, Washington, DC USA

**Keywords:** Anemia, Breastfeeding, Cost-benefit analysis, Nutrition, Stunting, Small for gestational age, Wasting

## Abstract

**Background:**

The world is not on track to reach the Sustainable Development Goal nutrition targets. We aimed to estimate the cost, impact and benefit-cost ratio from scaling up 15 evidence-based nutrition interventions among 125 countries and seven world regions.

**Methods:**

For each country, the Optima Nutrition model was used to project health outcomes over 2025–2034 for pregnant women (anemia, mortality) and children under five (stunting, wasting, anemia, small for gestational age births, children exclusively breastfed). The 15 interventions were identified through systematic reviews. Country-specific demographic, epidemiological and intervention coverage parameters were extracted from large population surveys, World Bank and Institute of Health Metrics and Evaluation estimates. A baseline scenario with current coverage of interventions maintained was compared to an investment scenario where intervention coverage was increased linearly to 90% over 2025 to 2029 and then maintained from 2030 to 2034. Country-specific intervention unit costs were estimated from a health system perspective and used to calculate 10-year intervention costs for each scenario. Health impacts for the 10-year cohort were converted to societal economic benefits from larger and more productive future workforces. Costs and economic benefits are presented in 2023 US$ with 3% per annum discounting.

**Results:**

The current coverage of interventions varied widely across countries but was generally low. Compared to the baseline, the investment scenario cost an average additional US$4.87–11.43 per pregnant woman and an average additional US$9.99–20.14 per child under five per annum, depending on world region. Across regions, this investment could reduce child deaths by 7–13%, maternal deaths by 5–9%, stunted children turning age five by 5–9%, wasting episodes by 2–4%, maternal anemia cases by 36–38%, child anemia cases by 16–23%, small for gestational age births by 46–52%, and could increase the number of infants exclusively breastfed by 10–29%. These improved outcomes translated to benefit-cost ratios of 13–54 across regions. The estimated financial needs, distribution of additional costs across interventions and return-on-investment for each country was dependent on population sizes and country-specific epidemiological and economic indicators.

**Conclusions:**

Scaling up evidence-based nutrition interventions could have substantive health and economic impacts. For every $1 invested there could be between $13 and $54 in economic benefits.

**Supplementary Information:**

The online version contains supplementary material available at 10.1186/s12939-026-02872-5.

## Introduction

Undernutrition drives some of the greatest inequities in health and is estimated to underly 45% of child deaths globally [1]. As well as increasing child mortality, undernutrition contributes significantly to impaired learning and future earning capacity, which can exacerbate poverty, perpetuate intergenerational disadvantage, and slow the economic development of low- and middle-income countries (LMICs).

In 2012, the World Health Assembly endorsed six global nutrition targets to be achieved by 2025, relating to child stunting, wasting and overweight, exclusive breastfeeding, low birthweight, and anemia in women of reproductive age (15–49 years) [2]. In 2015 these were incorporated into the Sustainable Development Goals [3] (under target 2.2, which calls for an end to all forms of malnutrition), and in 2023 were extended to 2030 targets [4] aiming to achieve, relative to 2012: a 50% reduction in the number of children under-five who are stunted; a reduction in child wasting to < 3% prevalence; a 50% reduction in anaemia in women of reproductive age; at least 70% exclusive breastfeeding of children under six months; and a 30% reduction in low birthweight [5]. Analyses have shown that despite improvements in these indicators over the last two decades, most countries are not on track to reach these targets [6].

There are a range of interventions available that have proven to be effective in addressing undernutrition, as summarized in WHO guidelines [7, 8] and multiple Lancet nutrition series (2008 [9], 2013 [10], 2019 [11], 2021 [12, 13]). Despite evidence of the potential impact that scaling up these interventions could have on achieving the nutrition targets [6, 10, 14, 15], to date their coverage remains low. A major barrier to scaling up nutrition interventions is their cost; however, countries may not realise how much undernutrition is already costing their economies through lost productivity and economic development. Evidence is needed to quantify the economic benefits that nutrition interventions can have to strengthen the case for investment in them.

In 2024, the World Bank developed an updated Investment Framework for Nutrition [16], expanding on the 2017 Investment Framework [15] with the latest evidence for interventions and policy measures to improve nutrition outcomes. The Investment Framework included a chapter estimating the cost, impact and return-on-investment for scaling up 15 evidence-based nutrition interventions across subsets of high burden or high priority countries (defined as the greatest absolute burden and greatest prevalence of undernutrition outcomes). These estimates were extrapolated globally using multiplier methods, based on relative burden of different nutritional outcomes.

While the Investment Framework has been critical for advocacy and obtaining funding commitments from donors, nutrition indicators vary widely across regions. For example, in 2024, West and Central Africa, Eastern and Southern Africa and South Asia had the highest percentage of children under five affected by stunting, about 32% in each region, compared to 23.2% globally [17]. South Asia also had the highest percentage of children under five affected by wasting, 14.1% compared to 6.6% globally [17]. These differences highlight the need to generate more granular economic analyses to better inform regional investment cases and to guide allocation of limited resources.

The aim of this study is to extend the global Investment Framework by providing regional estimates for the cost, impact and return-on-investment from scaling up packages of evidence-based nutrition interventions.

## Methods

### Model overview

The analysis uses the Optima Nutrition model, following the Investment Framework. Optima Nutrition is a mathematical compartmental model that projects health outcomes for a given setting under different investment and intervention scenarios.

The model tracks the number of women of reproductive age (15–49 years) in a population, who can become pregnant and give birth. After birth, children are tracked until five years of age across five age bands: <1 month, 1–5 months, 6–11 months, 12–23 months and 24–59 months. Children in each age-band are categorized by height-for-age (stunting) status, weight-for-height (wasting) status, anemia status, breastfeeding practice, and economic status (above or below the poverty line). Women of reproductive age are classified by anemia status.

Children exit the model either when they reach the age of 60 months or by death, which can happen at any age and by specific causes. The relative risks of dying from each cause are related to the child’s birth outcomes (pre-term birth and/or a child being born small for gestational age [SGA]) [18], breastfeeding [19, 20], height-for-age [21] and weight-for-height [21] status. Mortality is also tracked for pregnant women, with relative mortality risks related to anemia status [22].

Several risk factors for stunting in children are modelled: birth outcomes [23], stunting in a previous age-band [24] and incidence of diarrhea [25]. In addition, anemia in pregnant women is modelled to be a risk factor for sub-optimal birth outcomes [26, 27]; birth outcomes [28] and diarrhea incidence [29] are modelled to be risk factors for wasting; and sub-optimal breastfeeding is modelled to be a risk factor for diarrhea incidence [19].

**Fig. 1:**
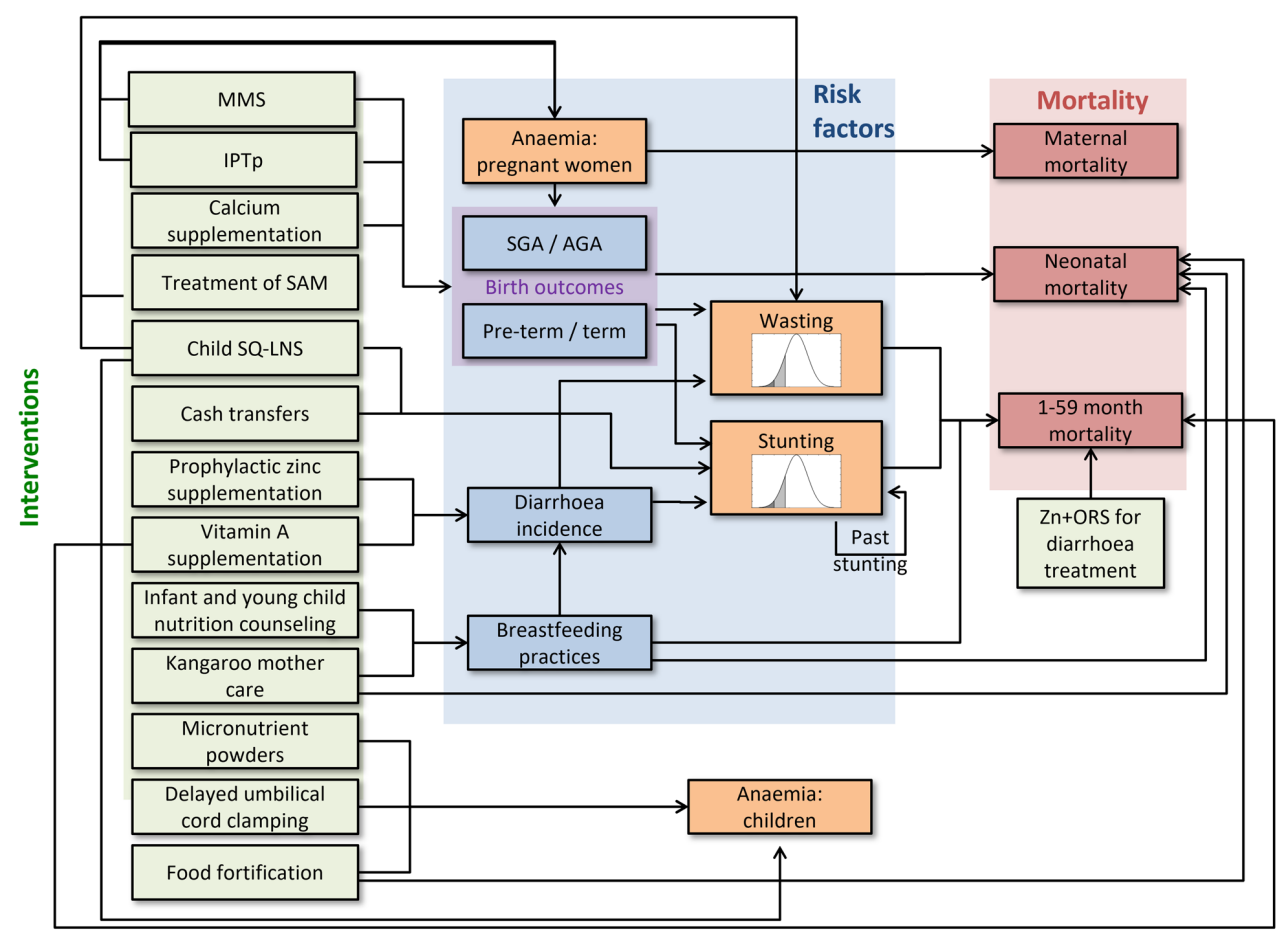
The relationship between interventions, risk factors and mortality. AGA, appropriate for gestational age; IPTp, intermitted presumptive treatment of malaria during pregnancy; MMS, multiple micronutrient supplements; Zn + ORS, zinc and oral rehydration solution; SAM, severe acute malnutrition; SGA, small for gestational age. Source: World Bank, Investment Framework for Nutrition 2024 [16]

In the model, interventions can improve nutritional outcomes directly or indirectly by reducing risk factors (Fig. 1). Changing the coverage of an intervention among its target population leads to changes in projected outcomes based global estimates of intervention effectiveness. For example, changes to breastfeeding practices, perhaps through better education, can directly reduce mortality and diarrhea incidence. Moreover, in the model this will also lead to an indirect reduction in mortality because a reduction in diarrhea incidence will lead to a reduction in stunting and wasting, which will subsequently further reduce mortality.

Interventions are applied as relative reductions in the different conditions or outcomes, based on effect estimates from the literature (as either relative risks, or odds ratios converted to relative risks, based on country-specific prevalence of conditions and baseline coverage). Interventions are applied as sequential relative risks, meaning they are not additive but assumed to be independent. It is also possible to apply pair-wise constraints on interventions; for example, children receiving small-quantity lipid-based nutrition supplements (SQ-LNS) are not eligible to receive zinc supplementation or micronutrient powders.

Optima Nutrition uses an economic model to translate the amount spent on an intervention to its estimated coverage. For each intervention, this requires a setting-specific input for the unit cost, as well as baseline coverage levels to inform potential scale-up.

Additional model details, including parameters, are in Supplementary Material 1, Appendix A.

### Settings

The modelling was conducted for 125 countries at the national level, and results are presented in aggregate by World Bank region (Supplementary Material 1, Appendix B): Africa West (AFW), Eastern and Southern Africa (AFE), Middle East and North Africa (MENA), East Asia and Pacific (EAP), South Asia (SA), Latin America and Caribbean (LAC), Eastern Europe and Centra Asia (EECA). Each country is modelled independently, and so heterogeneities emerge due to differences in baseline demographic, epidemiological and economic model input parameters.

Model inputs for each country are in Supplementary Material 2 and Supplementary Material 3.

### Interventions and effect estimates

The analysis included 15 interventions that were identified through systematic reviews [30] as part of the development of the World Bank Investment Framework for Nutrition (Fig. 1). For each intervention, the target population, effects, effect sizes are provided in Supplementary Material 1, Appendix C. Where intervention effects were not statistically significant, they were not included in the analysis.

Each region included either all 15 interventions or a subset of them (Supplementary Material 1, Appendix B), based on consultation with World Bank regional offices as to the applicability of interventions.

### Intervention costs

The unit cost for each intervention was estimated for each country from a health system perspective. Unit costs were sourced from the scientific literature, grey literature, previous analytical studies conducted by the World Bank, and global costs taken from the 2017 and 2024 Investment Frameworks [15, 16]. When a unit cost estimate was not available for a particular country, the mean from other countries in the same region were used provided three or more countries informed the regional average, otherwise a global estimate was used.

Unit costs were assumed to be constant with intervention coverage, because limited evidence is available to inform how unit costs may vary with scale (which may lead to underestimating costs at high coverage) or efficiencies when interventions are delivered as a package (which may lead to overestimation of costs). Country-specific assessments will be required to disaggregate costs by sector or delivery platform.

Costs are presented in 2023 US$, with 3% per annum discounting applied to future costs. Additional details on unit costs are available in Supplementary Material 1, Appendix D.

### Baseline intervention coverages

Baseline coverage estimates for nutrition and health interventions were primarily based on the most recent Demographic and Health Surveys (DHS), supplemented with proxy indicators and default values from the Lives Saved Tool (LiST) when DHS data were unavailable. Additional assumptions were applied for interventions lacking direct coverage data, such as food fortification and infant and young child nutrition counselling. Interventions not yet widely scaled were assigned a baseline coverage of zero. Full details on sources, assumptions, and methods used to derive baseline coverage can be found in Supplementary Material 1, Appendix E.

### Scenarios

For each country, two scenarios were compared over the 10-year period 2025–2034: (1) a baseline scenario, where intervention coverage (as a proportion of the target population) was maintained; and (2) an investment scenario, where intervention coverages were increased linearly from current levels to reach 90% coverage among their target populations in 2029, and maintained for 2030–2034.

### Epidemiological outcomes

For each scenario, the model projected outcomes for the number of deaths in children under five, maternal deaths, stunted children turning age five, episodes of wasting, cases of anemia in children under five, children exclusively breastfed, SGA births, and cases of maternal anemia.

### Economic benefits

The improved health and nutrition outcomes gained from scaling up interventions were translated into economic benefits in the form of increased potential income, from either increased workforce size (from deaths averted) or increased workforce productivity (from improved child development or reduced maternal anemia). Average income was based on country-specific gross domestic product (GDP) per capita, assuming 90% of lifetime earnings are realized [31], and adjusting for country- and age-specific all-cause mortality [32] among the children whose adverse outcomes were averted (since not all would survive their entire working lives). Country GDP was assumed to grow at 3% per annum, working age was assumed to be 18–65 years.

Lifetime economic benefits were considered for the cohorts who received interventions in the 2025–2034 period, with 3% per annum discounting applied to future economic benefits. Details of economic benefit calculations are in Supplementary Material 1, Appendix F.

### Uncertainty analyses

A multivariate probabilistic uncertainty analysis was undertaken to generate uncertainty intervals for impact, economic benefit and benefit-cost ratio estimates. For each country and scenario, the model was run 100 times using sampled parameter sets. Parameters were sampled for intervention effect sizes (uniformly from their uncertainty bounds; Supplementary Material 1, Appendix C), relationships between risk factors and outcomes in the model (uniformly from ± 10% as ranges were not available; Supplementary Material 1, Appendix A), intervention unit costs (uniformly from ± 25%, given greater variation and uncertainty is expected), and economic benefits inputs (uniformly from ± 25%, Supplementary Material 1, Appendix F). Central 95-percentiles were retained as uncertainty bounds.

### Sensitivity analyses

Univariate sensitivity analyses were run considering different discounting rates (costs and benefit, 0% or 5% versus 3%); different GDP growth assumptions (1% or 5% versus 3%); longer intervention scale-up period (10 years versus 5 years); higher unit costs (25% or 50% higher); baseline intervention coverage assumed to be 10% or 20% where data was missing versus 0%); GDP for each country was 10% higher or lower; and the impact of stunting on future productivity gains was halved.

Vitamin A supplementation is modelled to reduce mortality among 1–59-month children both directly and indirectly (i.e. via reduced diarrhoea, leading to reduced stunting, leading to reduce mortality), with the indirect effect being important to capture the benefits of vitamin A on stunting. To assess the extent to which this may double count some mortality benefits, a scenario was run without the indirect effect to measure the difference in mortality and associated economic outcomes.

## Results

### Baseline scenario

**Fig. 2:**
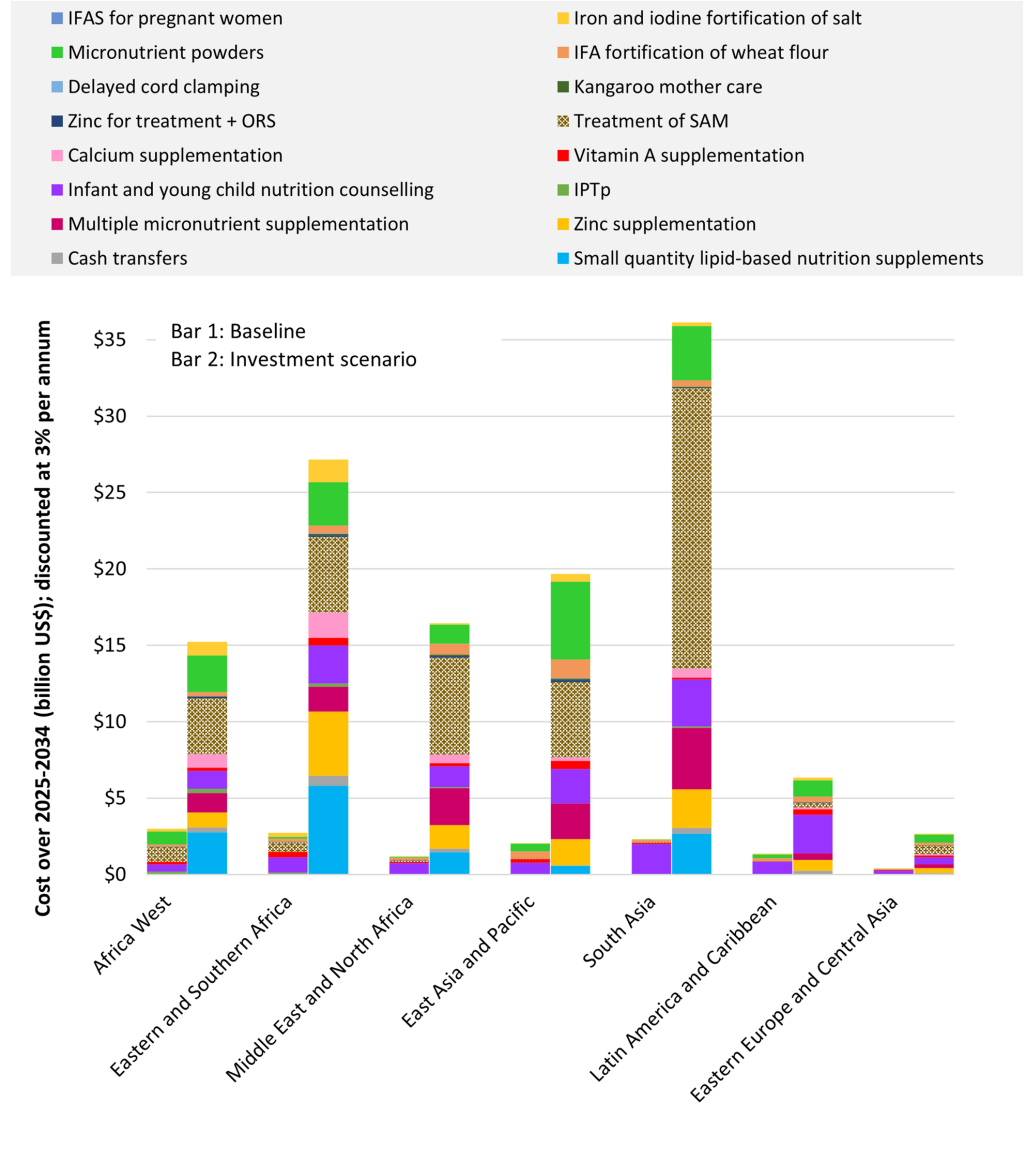
Total cost of the baseline and investment scenarios for each region over 2025–2034, by intervention. Costs are aggregated across countries included in each regional analysis, presented in 2023 US$ with future costs discounted at 3% per annum. IFAS: iron and folic acid supplementation; IPTp: intermitted presumptive treatment of malaria during pregnancy; ORS: oral rehydration solution; SAM: severe acute malnutrition

The current coverage of interventions varied widely across countries but was generally low (Appendix F). When aggregated at a regional level, the estimated cost of maintaining the current coverage of interventions over 2025–2034 ranged from US$382 million in Eastern Europe and Central Asia to US$3.00 billion in Africa West (Fig. 2 and Supplementary Material 1, Appendix G). The greatest proportion of current spending was for infant and young child nutrition counselling in all regions except Africa West, where it was treatment of severe acute malnutrition (SAM) (Supplementary Material 1, Appendix G).

Without any changes to intervention coverage, the model projected over 2025–2034 across the 125 countries and seven regions there could be 63 million deaths in children under five 383 million stunted children turning age five, 228 million episodes of wasting, 314 million cases of anemia in children under five, 18 million SGA births, 388 million cases of maternal anemia, 2.7 million maternal deaths, and 467 million children exclusively breastfed (Supplementary Material 1, Appendix G).

### Investment scenario

The additional cost over 2025–2034 to scale up the packages of evidence-based nutrition interventions ranged from US$2.29 billion in Eastern Europe and Central Asia to US$33.85 billion in South Asia (Table 1). These estimated financial needs depended on population sizes, the packages of interventions included for each region, and country-level epidemiological inputs determining eligibility for interventions (e.g. interventions targeted to those below the poverty line). The average additional cost per pregnant woman ranged from US$4.87 in Latin America and Caribbean to US$11.43 in South Asia, and the average additional cost per child under five per year ranged from US$9.99 in Eastern Europe and Central Asia to US$20.14 in South Asia (Table 1).

**Table 1:** Summary of health outcomes, costs and benefits across regions over 2025*–*2034. Regional values aggregate across countries included in analysis. Cost and economic benefits are in 2023 US$ with future costs and benefits discounted at 3% per annum. Values represent point estimates and 95% uncertainty bounds from multivariate probabilistic uncertainty analysis

Region	Africa West	Eastern and Southern Africa	Middle East and North Africa	East Asia and Pacific	South Asia	Latin America and Caribbean	Eastern Europe and Central Asia
Additional cost (billions)	$12.22 ($11.00 – $13.38)	$24.39 ($21.89 – $26.84)	$15.26 ($13.33 – $17.00)	$17.63 ($15.62 – $19.38)	$33.85 ($28.93 – $38.35)	$4.97 ($4.29 – $5.57)	$2.29 ($2.04 – $2.52)
*Average additional cost per pregnant woman*	$7.87 ($6.35 – $9.20)	$8.09 ($6.47 – $9.53)	$11.24 ($8.92 – $13.44)	$8.03 ($6.27 – $9.77)	$11.43 ($9.08 – $13.79)	$4.87 ($3.83 – $5.85)	$6.78 ($5.35 – $8.09)
*Average additional cost per child under five per year*	$11.25 ($9.91 – $12.56)	$15.82 ($13.97 – $17.62)	$13.45 ($11.43 – $15.22)	$13.35 ($11.48 – $14.86)	$20.14 ($16.70 – $23.21)	$10.36 ($8.79 – $11.71)	$9.99 ($8.62 – $11.00)
Health impacts							
*Child deaths averted*	1.42 M (7%) (1.12 M – 1.60 M)	1.71 M (10%) (1.54 M – 1.88 M)	1.16 M (12%) (1.04 M – 1.28 M)	0.31 M (8%) (0.28 M – 0.35 M)	1.28 M (13%) (1.15 M – 1.41 M)	0.13 M (8%) (0.11 M – 0.14 M)	0.05 M (9%) (0.04 M – 0.05 M)
*Stunting cases averted*	4.12 M (8%) (3.10 M – 6.97 M)	7.20 M (8%) (6.48 M – 7.92 M)	4.05 M (7%) (3.64 M – 4.45 M)	3.41 M (5%) (3.07 M – 3.75 M)	6.13 M (6%) (5.51 M – 6.74 M)	0.95 M (9%) (0.86 M – 1.05 M)	0.24 M (7%) (0.22 M – 0.27 M)
*Wasting episodes averted*	1.13 M (4%) (0.73 M – 1.25 M)	1.39 M (4%) (1.25 M – 1.52 M)	1.08 M (4%) (0.97 M – 1.19 M)	0.99 M (3%) (0.89 M – 1.09 M)	2.08 M (2%) (1.87 M – 2.29 M)	0.09 M (3%) (0.08 M – 0.10 M)	0.04 M (2%) (0.04 M – 0.05 M)
*Child anemia cases averted*	10.97 M (20%) (9.43 M – 12.13 M)	18.14 M (23%) (16.33 M – 19.95 M)	10.12 M (21%) (9.11 M – 11.14 M)	4.61 M (18%) (4.14 M – 5.07 M)	17.85 M (22%) (16.06 M – 19.63 M)	2.36 M (16%) (2.12 M – 2.59 M)	1.46 M (19%) (1.31 M – 1.61 M)
*Small for gestational age births averted*	0.65 M (52%) (0.31 M – 0.78 M)	1.16 M (49%) (1.04 M – 1.27 M)	1.66 M (47%) (1.49 M – 1.82 M)	0.65 M (47%) (0.59 M – 0.72 M)	4.36 M (48%) (3.92 M – 4.79 M)	0.24 M (46%) (0.21 M – 0.26 M)	0.11 M (47%) (0.10 M – 0.12 M)
*Additional children exclusively breastfed*	13.92 M (29%) (11.27 M – 16.83 M)	19.33 M (17%) (17.40 M – 21.27 M)	9.59 M (17%) (8.63 M – 10.54 M)	18.57 M (23%) (16.71 M – 20.42 M)	13.52 M (10%) (12.17 M – 14.88 M)	6.36 M (27%) (5.73 M – 7.00 M)	1.99 M (20%) (1.79 M – 2.18 M)
*Maternal anemia cases averted*	24.96 M (38%) (19.26 M – 28.23 M)	27.14 M (36%) (24.42 M – 29.85 M)	21.27 M (37%) (19.14 M – 23.40 M)	25.40 M (37%) (22.86 M – 27.94 M)	34.43 M (37%) (30.99 M – 37.87 M)	7.01 M (36%) (6.31 M – 7.71 M)	3.55 M (36%) (3.19 M – 3.90 M)
*Maternal deaths averted*	77.0k (7%) (62.0k – 85.4k)	46.6k (6%) (42.0k – 51.3k)	23.2k (9%) (20.8k – 25.5k)	11.6k (7%) (10.4k – 12.7k)	18.8k (7%) (17.0k – 20.7k)	3.1k (5%) (2.8k – 3.5k)	0.4k (5%) (0.4k – 0.4k)
Economic benefits (in billion US$)							
Total Economic benefits	$280.76 ($207.46 – $396.76)	$321.67 ($234.33 – $473.53)	$302.29 ($206.52 – $389.38)	$950.44 ($597.35 – $1,277.33)	$568.15 ($386.60 – $744.28)	$243.19 ($168.22 – $333.45)	$62.93 ($44.02 – $87.97)
*Child deaths averted*	$90.28 (32%) ($60.23 – $103.73)	$74.06 (23%) ($47.47 – $88.21)	$83.27 (28%) ($49.14 – $98.31)	$69.23 (7%) ($41.25 – $83.94)	$124.91 (22%) ($75.61 – $145.24)	$34.84 (14%) ($20.57 – $41.76)	$11.52 (18%) ($6.88 – $14.17)
*Stunting cases averted*	$171.37 (61%) ($121.45 – $285.09)	$231.21 (72%) ($163.80 – $382.24)	$201.92 (67%) ($132.96 – $286.20)	$818.65 (86%) ($492.66 – $1,130.85)	$394.80 (69%) ($268.28 – $567.07)	$187.03 (77%) ($123.89 – $274.11)	$45.48 (72%) ($30.06 – $69.81)
*Maternal deaths averted*	$2.15 (0.8%) ($1.47 – $2.54)	$0.96 (0.3%) ($0.63 – $1.15)	$0.22 (0.1%) ($0.15 – $0.26)	$1.26 (0.1%) ($0.84 – $1.51)	$0.48 (0.1%) ($0.33 – $0.58)	$0.35 (0.1%) ($0.23 – $0.42)	$0.04 (0.1%) ($0.03 – $0.05)
*Maternal anemia averted*	$0.31 (0.1%) ($0.18 – $0.42)	$0.22 (0.1%) ($0.13 – $0.31)	$0.08 (0.0%) ($0.05 – $0.11)	$1.12 (0.1%) ($0.65 – $1.54)	$0.29 (0.1%) ($0.17 – $0.40)	$0.23 (0.1%) ($0.13 – $0.32)	$0.08 (0.1%) ($0.05 – $0.11)
*Additional breastfeeding*	$5.17 (1.8%) ($2.72 – $8.17)	$3.78 (1.2%) ($2.00 – $5.94)	$3.48 (1.2%) ($1.84 – $5.49)	$35.30 (3.7%) ($18.72 – $55.15)	$6.24 (1.1%) ($3.31 – $9.74)	$9.17 (3.8%) ($4.82 – $14.61)	$1.63 (2.6%) ($0.86 – $2.58)
*Small for gestational age births averted*	$1.94 (0.7%) ($0.81 – $2.87)	$2.08 (0.6%) ($0.63 – $3.19)	$4.05 (1.3%) ($1.36 – $6.17)	$8.52 (0.9%) ($2.79 – $12.98)	$17.85 (3.1%) ($6.29 – $27.20)	$2.76 (1.1%) ($0.91 – $4.19)	$0.90 (1.4%) ($0.29 – $1.36)
*Child anemia averted*	$9.54 (3.4%) ($5.32 – $14.39)	$9.36 (2.9%) ($5.22 – $14.16)	$9.26 (3.1%) ($5.12 – $14.05)	$16.36 (1.7%) ($8.97 – $24.74)	$23.57 (4.1%) ($13.06 – $35.77)	$8.81 (3.6%) ($4.86 – $13.36)	$3.28 (5.2%) ($1.80 – $4.97)
Benefit-cost ratio	23 (17–33)	13 (10–19)	20 (14–27)	54 (34–77)	17 (11–23)	49 (35–70)	27 (20–40)

The largest proportion of the additional costs were for treatment of SAM (across regions 18–54% of additional costs, except for Latin America and Caribbean where it was only 6% due to lower wasting prevalence). Aside from this, the distribution of additional costs across interventions differed by region, depending on unit costs, baseline coverages and epidemiological indicators (Supplementary Material 1, Appendix G).

**Fig. 3:**
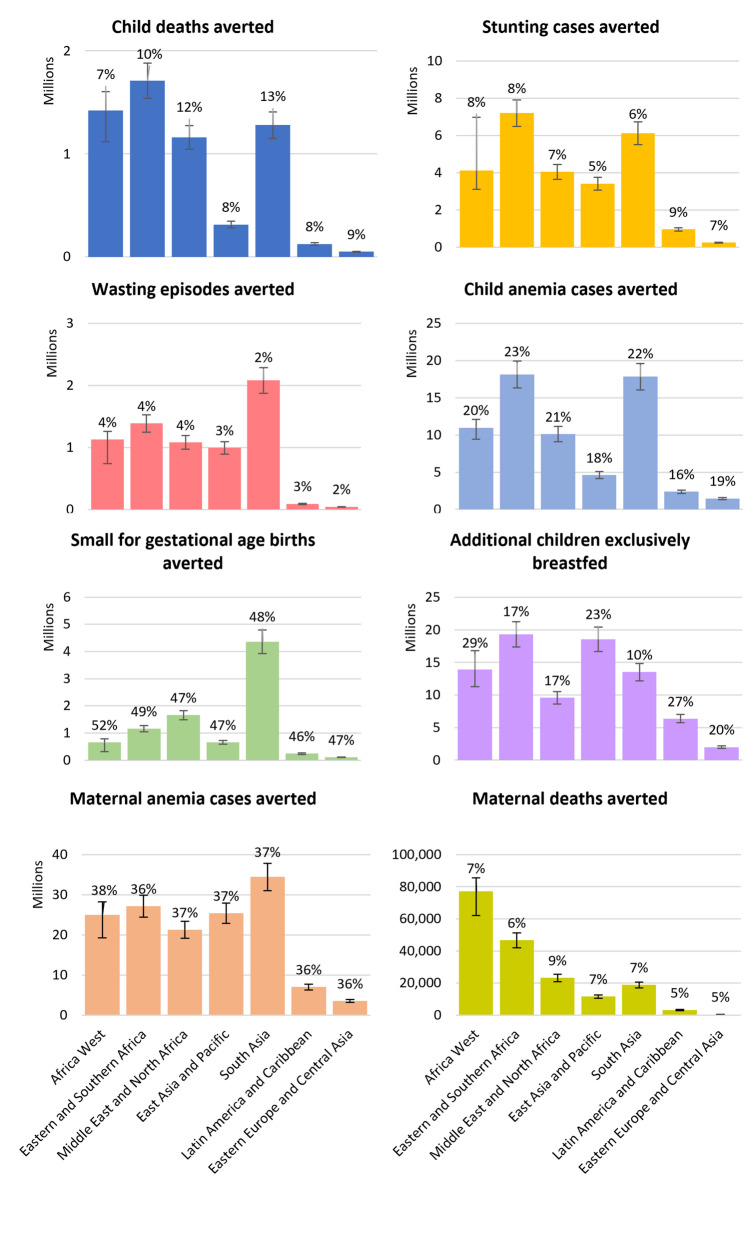
Cumulative health impact (and percentage reduction compared to baseline) of scaling up interventions from baseline coverage to 90% coverage over 2025–2029 and maintaining 90% coverage from 2030–2034; aggregated across countries in each region. Child deaths averted (row 1; left), stunting cases averted (row 1; right), wasting episodes averted (row 2, left), child anemia cases averted (row 2, right), small for gestational age births averted (row 3, left), additional children exclusively breastfed (row 3, right), maternal anemia cases averted (row 4; left) and maternal deaths averted (row 4; right). The percentages represent the change compared to the baseline

Across regions, this investment was estimated reduce child deaths by 7–13%, maternal deaths by 5–9%, stunted children turning age five by 5–9%, wasting episodes by 2–4%, maternal anemia cases by 36–38%, child anemia cases by 16–23%, SGA births by 46–52%, and to increase the number of infants exclusively breastfed by 10–29% (Fig. 3; Table 1).

**Fig. 4:**
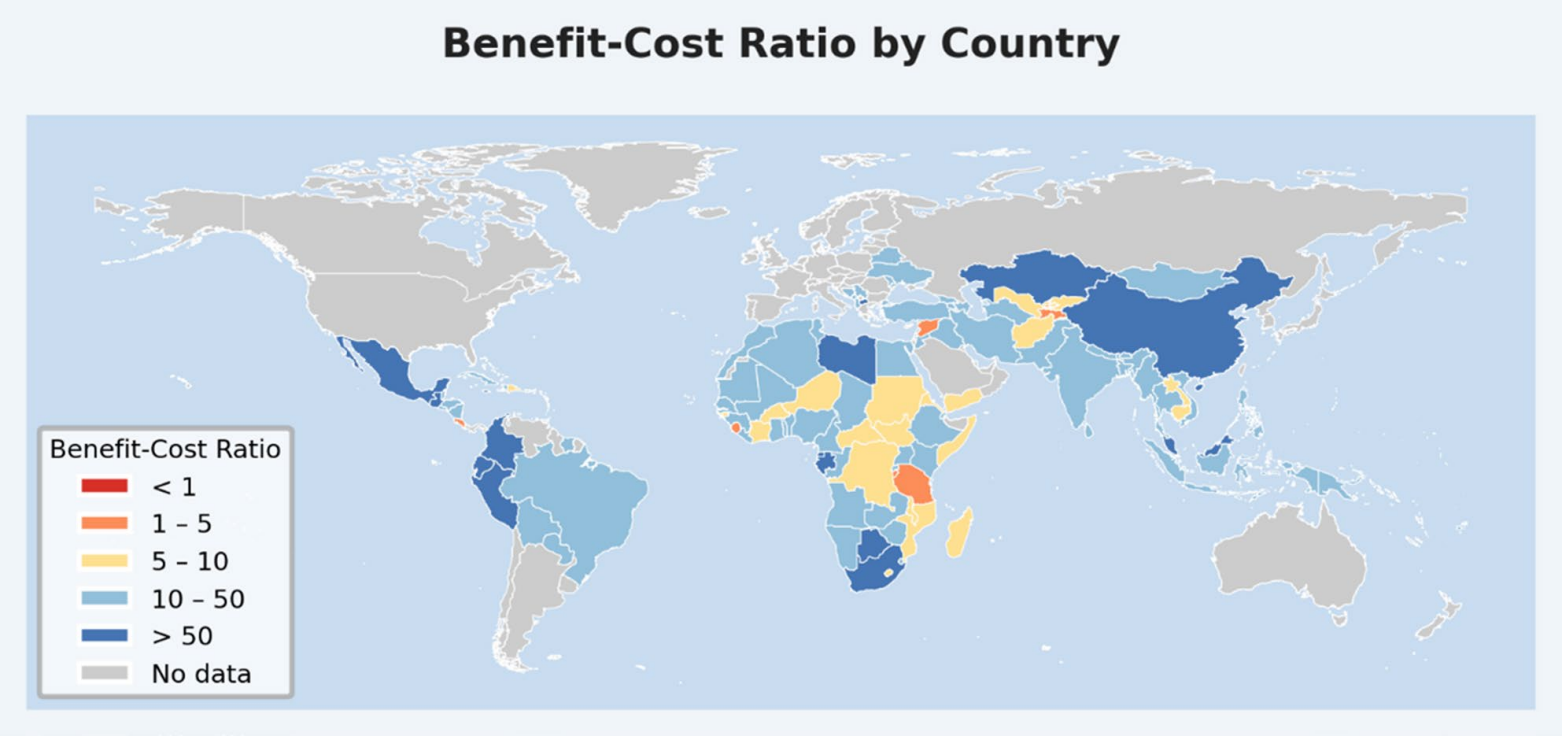
Estimated benefit-cost ratio for the investment scenario compared to the baseline scenario for each country. Countries represented in grey were not included in the analysis. Costs and benefits were discounted at 3% per annum

These health impacts were estimated to translate to economic benefits ranging from $63 billion in Eastern Europe and Central Asia to $950 billion in East Asia and Pacific, and across regions resulted in benefit-cost ratios between 13 and 54 (Table 1). Return-on-investment varied substantially by country (Fig. 4), related to country-specific epidemiological and economic indicators.

Country-level results are in Supplementary Material 4.

### Sensitivity analyses

The benefit-cost ratios for each region varied as expected when model assumptions were changed (Table 2): that is, increasing with lower discounting, higher GDP or higher GDP growth assumptions. Halving the productivity impact of stunting had a large influence on benefit-cost ratios, as stunting contributes the largest proportion of overall economic benefits (ranging from 61 to 86% of benefits across regions). Removing the effect of vitamin A supplementation on diarrhoea led to a reduction in the benefit-cost ratio. The impacts on other model outcomes are in Supplementary Material 4.

**Table 2:** Summary of univariate sensitivity analysis, showing regional benefit-cost ratios under different parameter assumptions. Full set of outcomes are provided in Supplementary Material 4

Region	Africa West	Eastern and Southern Africa	Middle East and North Africa	East Asia and Pacific	South Asia	Latin America and Caribbean	Eastern Europe and Central Asia
Main analysis	23	13	20	54	17	49	27
Discounting of 0% (versus 3%)	83	47	72	199	62	180	101
Discounting of 5% (versus 3%)	11	6	9	25	8	23	13
GDP growth of 1% (versus 3%)	10	6	8	22	7	20	11
GDP growth of 5% (versus 3%)	58	33	50	137	43	124	69
Unit costs increased by 25%	18	11	16	43	13	39	22
Unit costs increased by 50%	15	9	13	36	11	33	18
Scale-up period of 10 years (versus five years)	23	13	20	54	16	49	28
Baseline intervention coverage assumed to be 10% where data is missing (versus 0%)	23	13	20	56	17	50	28
Baseline intervention coverage assumed to be 20% where data is missing (versus 0%)	23	14	21	58	18	52	29
GDP 10% higher	25	15	22	59	18	54	30
GDP 10% lower	21	12	18	49	15	44	25
Impact of stunting on future productivity halved	16	8	13	31	11	30	18
Impact of vitamin A supplementation of diarrhoea removed.	21	12	17	34	16	32	20

## Discussion

The 2024 World Bank Investment Framework for Nutrition identified 15 evidence-based interventions for reducing undernutrition. This study estimated that across seven world regions, scaling up packages of these interventions to 90% coverage over 2025–2034 would require an average additional US$4.87–11.43 per pregnant woman and an average additional US$9.99–20.14 per child under five per annum. This investment could reduce child deaths by 7–13%, maternal deaths by 5–9%, stunted children turning age five by 5–9%, wasting episodes by 2–4%, maternal anemia cases by 36–38%, child anemia cases by 16–23%, SGA births by 46–52%, and could increase the number of infants exclusively breastfed by 10–29%. Every dollar invested could return between 13 and 54 dollars in economic benefits across regions due to larger and more productive workforces.

The relative costs of scaling up different interventions does not necessarily reflect their relative cost-effectiveness. For example, some interventions may have lower unit costs than others meaning less investment is required to achieve high coverage, and these less expensive interventions may also have greater effect sizes leading to a lower costs per case averted. Previous work [16] using the Optima Nutrition model has identified that among the 15 interventions considered, the most cost-effective for stunting were vitamin A supplementation and cash transfers (with behaviour change communication); for wasting were vitamin A supplementation, zinc supplementation, and SQ-LNS; for child anemia were delayed cord clamping at birth and micronutrient powders; for breastfeeding were infant and young child nutrition counselling and kangaroo mother care; and for maternal anemia were intermittent preventive treatment of malaria in pregnancy and multiple micronutrient supplementation. However, policy makers and nutrition planners are likely to want to achieve progress on all of these indicators together. When considering multiple objectives, optimisation or other analyses would be required to determine which sub-packages of interventions should be prioritised if only limited investment were available. Considering multiple objectives together would also capture the benefits of interventions that impact multiple outcomes (e.g. SQ-LNS).

This study expands the global estimates in the 2024 World Bank Investment Framework for Nutrition [16] by considering regional estimates and tailored packages of interventions. The results show heterogeneity between regions in terms of cost, impact and economic benefits, driven by many factors including differences in demographics, epidemiology, unit costs, and economic indicators. Moreover, the results for each region are themselves aggregated over many heterogeneous countries (with country-specific results in Supplementary Material 4). For example, in the Latin America and Caribbean region, the prevalence of stunting varies widely across countries: from 43.5% in Guatemala and 1.6% in Chile [17]. The results in this study are useful for informing regional priorities, and while the country-specific results are not validated they may form a useful starting point for more detailed national or sub-national analyses led by country teams, based on local data rather than global datasets.

Many interventions could not be included in the analysis due to insufficient quantitative effect size estimates. Optima Nutrition is a global model and as such relies on a high threshold of evidence for interventions and/or risk factors to be included (i.e. typically meta-analyses of randomised controlled trials). This means that some interventions, particularly those impacting risk areas not represented in Fig. 1, may be being overlooked where trial data are unpublished or no meta-analysis exists because few published trials are available. When interpreting results, it is important to note that the interventions in this study are not exhaustive, and therefore the total impact estimated represent a fraction of what may be possible. Further work is required to continue to quantify the benefits of other interventions on nutrition outcomes.

More work is required to understand the unit costs of scaling up the nutrition interventions in different settings. Unit costs for this study were based on global reviews of the academic and grey literature, World Bank costing analyses, and other available country estimates. When reviewing and collating this data, many gaps were identified. While some costing studies could be identified for selected interventions in selected countries, nothing is currently available at a global level. Country-specific costing studies could more precisely account for staffing, infrastructure, logistic and other overhead costs and improve the accuracy of cost and return-on-investment estimates. We also assumed that the unit costs of interventions would remain constant with scale, which may not be the case as economies of scale may reduce marginal costs as coverage increases and saturation effects may decrease marginal costs as coverage becomes high.

There are some limitations to be considered when interpreting these results. First, economic benefits may be underestimated as we have calculated economic benefits as increased potential income, which is a more conservative approach than using Value of Statistical Life methods; and long-term benefits of improved undernutrition on noncommunicable diseases are not included. On the other hand, the economic benefits may be overestimated because secular improvements may reduce rates of disease and malnutrition in the absence of interventions. Second, there are limitations to the model structure. For example, the model is based on the risk factor structure / causal pathway outlined in Fig. 1 based on evidence available to support and quantify each relationship, but some of the evidence is only associative rather than causal. Third, population and epidemiological data inputs came largely from global data sets, and for some countries, this required imputing regional values or using modelled data where estimates were missing. Fourth, coverage estimates were not available for some interventions, which were assumed to be zero in the baseline. This may underestimate baseline costs, and overestimate investment requirements and total impact. Fifth, the aim of this study was to produce regional estimates for the return-on-investment of these interventions, but even within each region, the results for individual countries vary widely. Further work with validated country-level data could consider the main drivers of these heterogeneities, including the proportion that is explained by demography, baseline investments or economic factors. Finally, it is unclear how feasible it would be to scale up some of these interventions within five years. However, potentially, these results can support policy makers to at least increase investment in maternal and child nutrition.

While implementation and scaling challenges of evidence-based nutrition interventions have been previously documented [30], the current reductions in aid budgets from Western donor countries is perhaps the largest threat to achieving the proposed investments to address malnutrition. Systemic barriers such as reliance on external financing resources, limited prioritization of nutrition within policies and programs, and limited fiscal space, underscore the urgency for a new narrative around nutrition financing, including a reassessment of prioritization and nutrition funding methods [33], as well as a targeted work in leveraging multi-sectoral entry points.

## Conclusion

Scaling up evidence-based nutrition interventions could have substantive health and economic impacts. For every $1 invested, there could be between $13 and $54 in economic benefits.

## Electronic Supplementary Material

Below is the link to the electronic supplementary material.

Supplementary Material 1

Supplementary Material 2

Supplementary Material 3

Supplementary Material 4

## Data Availability

No datasets were generated or analysed during the current study.

## Abbreviations

DHS: Demographic and health surveys
GDP: Gross domestic product
LiST: Lives saved tool
LMIC: Low- and middle-income countries
SAM: Severe acute malnutrition
SGA: Small for gestational age
SQ-LNS: Small-quantity lipid-based nutrition supplements
WHO: World Health Organization
