# Supplementary material for: Scaling up evidence-based interventions to reduce maternal and child undernutrition in 125 countries: a cost-benefit analysis
Source: Int J Equity Health. 2026 May 12;25:163. doi: 10.1186/s12939-026-02872-5 (PMC13335392; doi:10.1186/s12939-026-02872-5)
Supplement: Supplementary file 1 — Supplementary Material 1 [file 12939_2026_2872_MOESM1_ESM.pdf]

Appendix A. Optima Nutrition model

Overview of populations and risk factors

The Optima Nutrition model tracks the number of women of reproductive age (15-49 years) in a population, who can become pregnant and give birth. After birth, children are tracked until five years of age across five age bands: <1 month, 1-5 months, 6-11 months, 12-23 months and 24-59 months (**Figure 1**).

Children in each age-band are categorized by height-for-age (stunting) status, weight-for-height (wasting) status, anemia status, breastfeeding practice, and economic status (above or below the poverty line). Women of reproductive age are classified by anemia status.

Children exit the model either when they reach the age of 60 months or by death, which can happen at any age. Children in the < 1 month age-band can die due to diarrhea, pneumonia, meningitis, asphyxia, sepsis, prematurity and "other" causes, while children in all other age bands can die of diarrhea, pneumonia, measles and "other" causes ("other" causes is used to capture, and match to, population statistics of known overall mortality rates for the given application context). The relative risks of dying from each cause are related to the child's breastfeeding, height-for-age, weight for height, and anemia status. Mortality is also tracked for pregnant women, who can die from antepartum hemorrhage, intrapartum hemorrhage, postpartum hemorrhage, hypertensive disorders, sepsis, abortion, embolism, other direct causes and other indirect causes. The relative risks of pregnant women dying from hemorrhage are related to their anemia status.

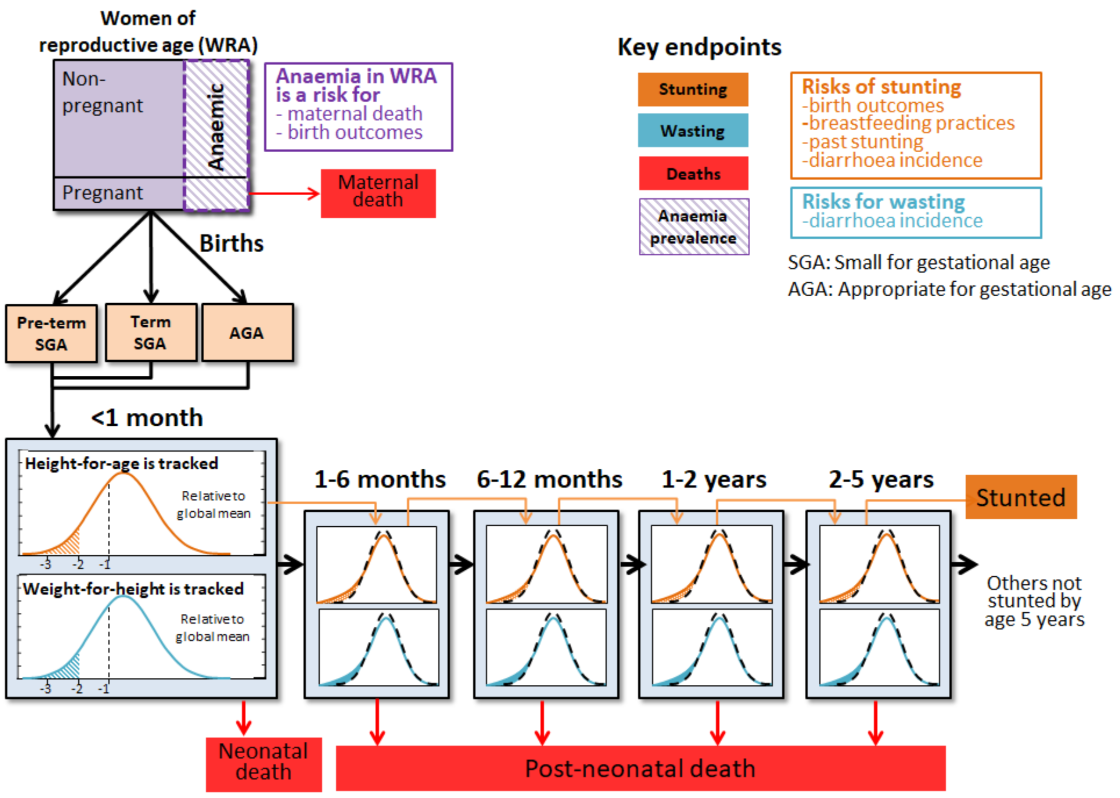

Figure 1: Populations within the Optima Nutrition model. AGA = appropriate for gestational age; SGA = small for gestational age; WRA = women of reproductive age

Several risk factors for stunting in children are modelled: birth outcomes (pre-term birth and/or a child being born small for gestational age [SGA]), stunting in a previous age-band and incidence of diarrhea (**Figure 2**). In addition, anemia in pregnant women is modelled to be a risk factor for sub-optimal birth outcomes; birth outcomes and diarrhea incidence are modelled to be risk factors for wasting; and sub-optimal breastfeeding is modelled to be a risk factor for diarrhea incidence.

In the model, interventions can improve nutritional outcomes directly or indirectly by reducing risk factors. For example, **Figure 2** shows that changes to breastfeeding practices, perhaps through better education, can directly reduce mortality and diarrhea incidence. Moreover, in the model this will also lead to an indirect reduction in mortality because a reduction in diarrhea incidence will lead to a reduction in stunting and wasting, which will subsequently further reduce mortality. Changing the coverage of an intervention among its target population leads to changes in projected outcomes based global estimates of intervention effectiveness.

Optima Nutrition uses an economic model to translate the amount spent on an intervention to its estimated coverage. For each intervention, this requires a setting-specific input for the unit cost.

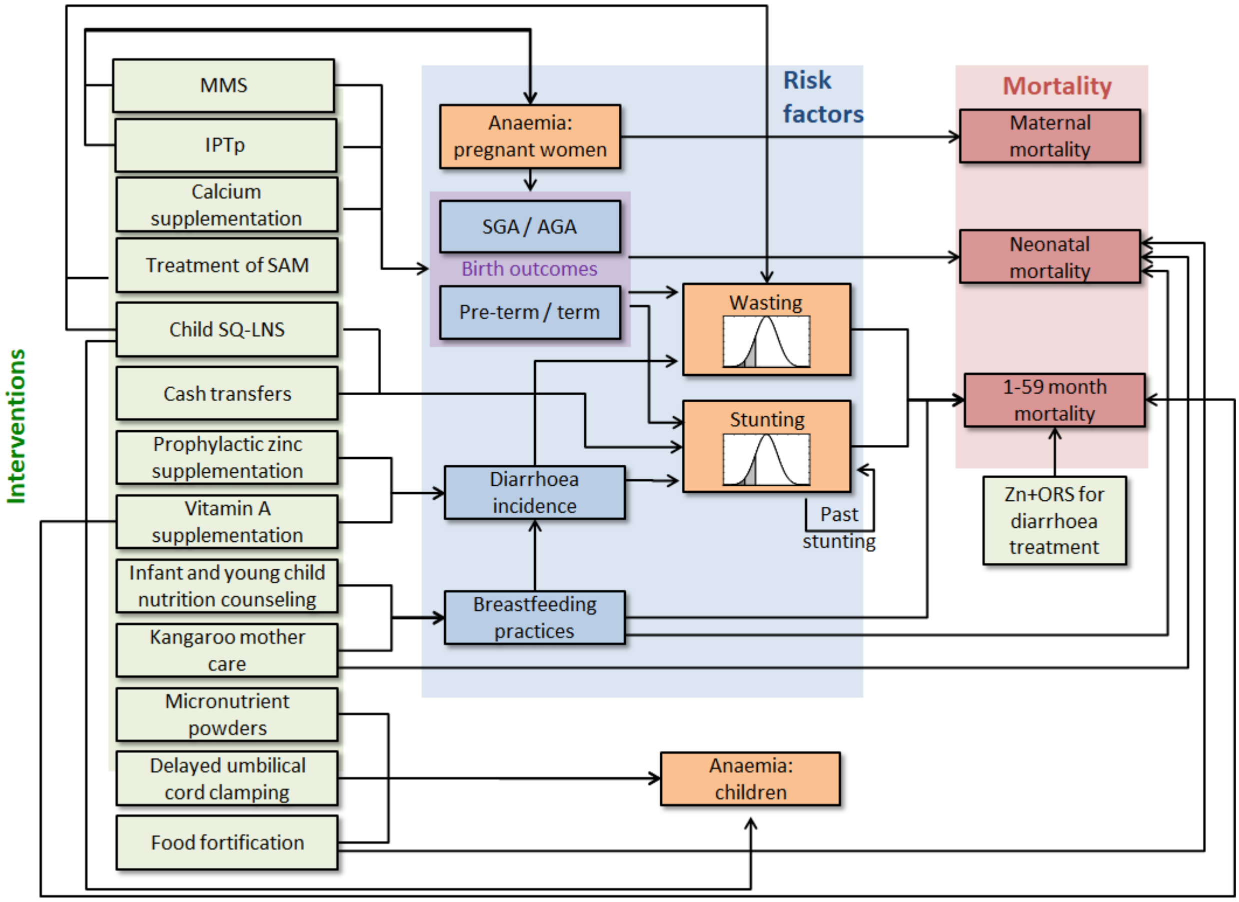

Figure 2: The relationship between interventions, risk factors and mortality AGA = appropriate for gestational age; IPTp = intermittent preventive treatment of malaria during pregnancy; MMS = multiple micronutrient supplements; SAM = severe acute malnutrition; SGA = small for gestational age; SQ-LNS = small-quantity lipid-based nutrient supplements.

Interventions are applied as relative reductions in the different conditions or outcomes, based on effect estimates from the literature (as either relative risks, or odds ratios converted to relative risks, based on country-specific prevalence of conditions and baseline coverage). Interventions are applied as sequential relative risks, meaning they are not additive but assumed to be independent. It is also possible to apply pair-wise constraints on interventions; for example, children receiving SQ-LNS are not eligible to receive zinc supplementation or micronutrient powders.

Modelling stunting using Optima Nutrition

- The model divides children in each age-band into four height-for-age categories, based on WHO criteria (**Figure 3**), with the two lowest categories (severe and moderate) being considered stunting:
  - Severe: < -3 standard deviations below the median height-for-age of the WHO reference population
  - Moderate: < -2 & >= -3 standard deviations below the median height-for-age of the WHO reference population
  - Mild: < -1 and >= -2 standard deviations below the median height-for-age of the WHO reference population
  - Normal: >= -1 standard deviation below the median height-for-age of the WHO reference population
- Risk factors for stunting are suboptimal birth outcomes (pre-term birth and/or a child being born SGA), stunting in a previous age-band, suboptimal feeding practices (age-appropriate breastfeeding and complementary foods), and incidence of diarrhea (**Figure 2**).
- Stunting increases the risk of mortality for children who have diarrhea, pneumonia, measles and other illnesses.
- Odds ratios and relative risks are model inputs, can be changed, and have defaults based on the literature.

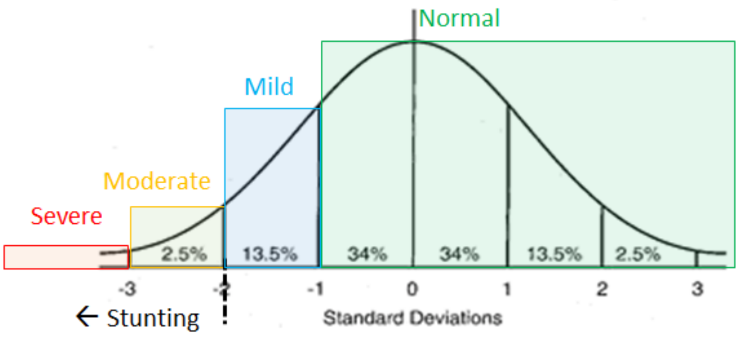

Figure 3: Categorization of the height-for-age distribution among children in the Optima Nutrition model. The model divides children in each age band into four height-for-age categories, based on World Health Organization criteria. Children in the two lowest categories (severe and moderate) are considered to be stunted.

Modelling wasting using Optima Nutrition

- The weight-for-height distribution is tracked for children in each age band (**Figure 4**, just like for stunting). Children are divided into four categories:
    - Severe acute malnutrition (SAM): < -3 standard deviations below than the median weight-for-height of the WHO reference population
    - Moderate acute malnutrition (MAM): < -2 and >= -3 standard deviations below than the median weight-for-height of the WHO reference population
    - Mild acute malnutrition: < -1 and >= -2 standard deviations below than the median weight-for-height of the WHO reference population
    - Normal: >= -1 standard deviation below than the median weight-for-height of the WHO reference population
- Children are considered to be "wasted" if they are in the SAM or MAM categories.
- Wasting is modelled as an incident (short-duration) condition:
    - As opposed to stunting, where being stunted in one age band increases the risk of being stunted in the next, wasting distributions are independent in each age band – this means that the distribution (i.e. prevalence) of wasting in a given time period does not affect the distribution of wasting in subsequent periods.
- Wasting increases the risk for mortality for children who have diarrhoea, pneumonia, measles and other illnesses
- Diarrhoea incidence and birth outcomes are risk factors for wasting (**Figure 2**):
    - Reducing diarrhoea incidence can lead to reductions in wasting
    - Improvements in birth outcomes (term/pre-tern; appropriate for gestational age [AGA]/SGA) can lead to reduced wasting

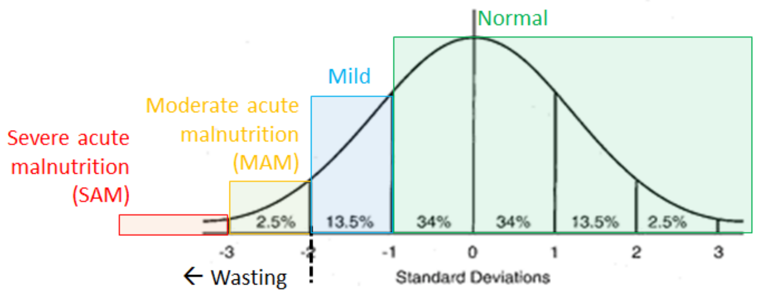

Figure 4: Categorization of the weight-for-height distribution among children in the Optima Nutition model. The model divides children in each age band into four weight-for-height z score categories. Children in the two lowest categories (SAM and MAM) are considered to be wasted. MAM = moderate acute malnutrition; SAM = severe acute malnutrition.

Figure 5 shows the dynamics of the wasting model within each age band:

- Children enter the age band (shown from the left), and will be classified as SAM, MAM, mild or normal according to the prevalence of these states from the data

- Children in the mild and normal categories can develop MAM (incidence of MAM)
- Children with MAM can deteriorate to SAM (incidence of SAM)
- When they are in MAM or SAM categories, children have increased risks of death
- Children can recover from the SAM and MAM categories due to treatment.
- The incidence rates, probabilities of death and average duration spent in SAM and MAM are calibrated to match country-specific data on prevalence, mortality and treatment numbers.

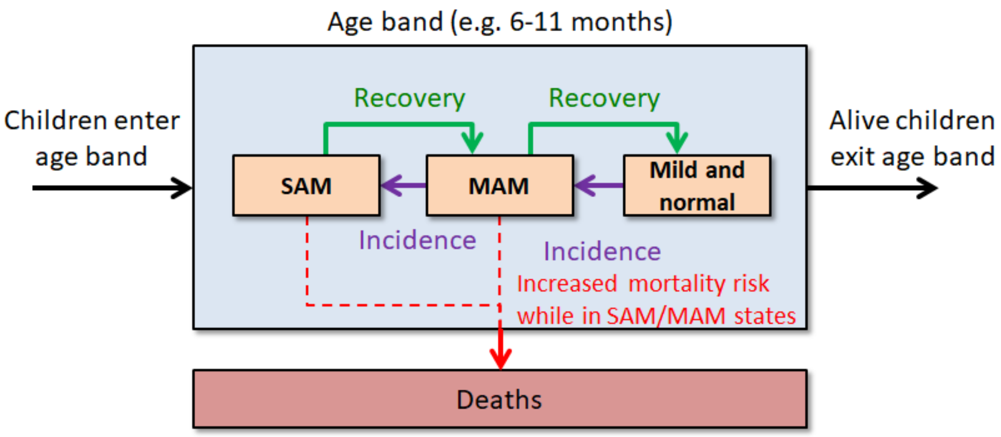

Figure 5: Wasting is considered to be an acute condition within the model. The model tracks the incidence of severe acute malnutrition (SAM) and moderate acute malnutrition (MAM) within each age band, allowing children to cycle between categories.

Modelling anemia using Optima Nutrition

Each population in the model is stratified by anemia status: anemic (mild, moderate, or severe) or not anemic (**Figure 6**). The model also includes a setting-specific input for the fraction of anemia that is severe.

- Anemia in pregnant women is modelled as a risk factor for maternal mortality (e.g. due to risk of hemorrhage)
- Anemia in pregnant women is also modelled to be a risk factor for suboptimal birth outcomes
    - This can affect stunting, which in turn can affect mortality in children.

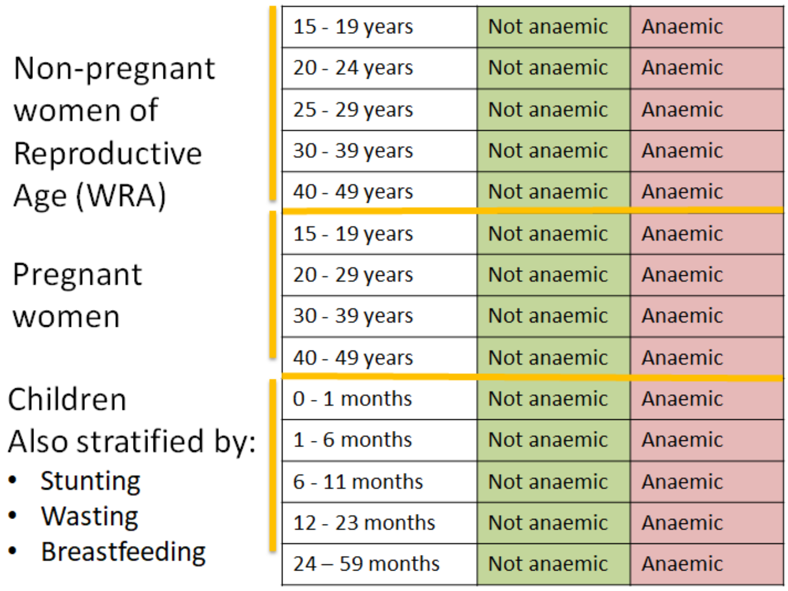

Figure 6: Summary of model populations. Each population is stratified by anemia status (anemic or not anemic). WRA = women of reproductive age

Mortality risk factors

Birth outcomes

Table 1: Relative risk ratios for neonatal mortality types by birth outcome (term / pre-term and appropriate for gestational age [AGA] / small for gestational age [SGA])

Birth outcome	Neonatal sepsis	Neonatal pneumonia	Neonatal asphyxia	Neonatal prematurity
Term AGA	Ref	Ref	Ref	Ref
Term SGA	2.07	2.07	2.07	1
Pre-term AGA	3.39	3.39	3.39	999.99
Pre-term SGA	11.89	11.89	11.89	999.99

Source: Katz et al. Lancet 2013 (1)

Stunting

Table 2: Relative risk ratios for 1–59-month-old mortality types, by height-for-age Z-score (HAZ) category

Age group	HAZ-status	Diarrhea	Pneumonia	Meningitis	Measles	Other
	None (HAZ-score >=-1)	Ref	Ref	Ref	Ref	Ref

1-59 months	Mild (HAZ-score >= -2 and < -1)	1.67	1.55	1	1	1
	Moderate (HAZ-score >= -3 and < -2)	2.38	2.18	1.86	2.79	1.86
	Severe (HAZ-score < -3)	6.33	6.39	3.01	6.01	3.01

Source: Olofin et al. PLOS One 2013 (2)

Wasting

Table 3: Relative risk ratios for 1–59-month-old mortality types, by weight-for-height Z-score (WHZ) category

Age group	Status	Diarrhoea	Pneumonia	Meningitis	Measles	Other
1-59 months	None (WHZ-score >= -1)	Ref	Ref	Ref	Ref	Ref
	Mild (WHZ-score >= -2 and < -1)	1.6	1.92	1.65	1	1.65
	MAM (WHZ-score >= -3 and < -2)	3.41	4.66	2.73	2.58	2.73
	SAM (WHZ-score < -3)	12.33	9.68	11.21	9.63	11.21

Source: Olofin et al. PLOS One 2013 (2)

Breastfeeding practices

Table 4: Relative risk ratios for mortality types, by breastfeeding practices and age group

Age group	Status	Neonatal diarrhoea / sepsis / pneumonia*	Diarrhoea†	Pneumonia^	Meningitis / measles / pertussis^
<1 month	Exclusive	Ref			
	Predominant	1.35			
	Partial	1.35			
	None	5.40			
1-5 months	Exclusive		Ref	Ref	
	Predominant		2.28	1.66	1.48
	Partial		4.62	2.50	2.84
	None		10.53	14.97	14.40
6-11 months	Partial		Ref	Ref	Ref
	None		1.47	1.92	3.69
12-23 months	Partial		Ref	Ref	Ref
	None		2.57	1.92	3.69

Sources: * NEOVITA Study Group 2016 with predominant / partial assuming late initiation (3); † Lamberti et al. BMC Public Health 2011 (4); ^ Lamberti et al. BMC Public Health 2013 (5).

Anemia

Table 5: Relative risks of maternal mortality types by anemia status.

Pregnant women age in years	Status	Antepartum haemorrhage	Intrapartum haemorrhage	Postpartum haemorrhage
15-49	Not anemic	Ref	Ref	Ref
15-49	anemic	10.675	10.675	10.675

Source: Heidkamp et al. J Nutr 2017(6). Applies only to the fraction who are severely anemic.

Birth outcomes

Impact of birth outcomes on stunting, wasting and anemia

Table 6: Odds ratios for stunting (HAZ-score <-2) and wasting (WHZ-score <-2), by birth outcome

	Odds ratio for condition if born:			
Condition	Term AGA	Term SGA	Pre-term AGA	Pre-term SGA
Stunting	Ref	5	6.4	46.5
Wasting	Ref	2.52	1.96	4.19

Sources: stunting LiST technical note (7); wasting Christian et al. Int J Epi 2013 (8) for low and middle income countries.

Odds of birth outcomes with maternal anemia

Table 7: Odds ratios for being born term / pre-term and appropriate for gestational age [AGA] / small for gestational age [SGA] if mother is anemic

Condition	Term SGA^	Pre-term AGA†	Pre-term SGA^
No maternal anemia	Ref	Ref	Ref
Maternal anemia	1.53	1.32	1.53

Sources: ^ Kozuki et al Journal of Nutrition 2012 (9); † Xiong et al. American Journal of Perinatology 2000 (10).

L.7 Diarrhea

Table 8: Relative risk of diarrhea by breastfeeding status

Age in months	<1 month	1-5 months	6-11 months	12-23 months

Exclusive	Ref	Ref		
Predominant	1.26	1.26		
Partial	1.68	1.68	Ref	Ref
None	2.65	2.65	2.07	2.07

Source: Lamberti et al BMC Public Health 2011 (4).

Table 9: Odds ratios for stunting, wasting and anemia as diarrhea incidence increases.

Condition	Age band	Odds ratio for every additional episode	Source
Stunting	0-59 months	1.025	Checkley et al. Int J Epi 2008 (11)
Wasting	0-59 months	1.024	Troeger et al Lancet 2018 (12)*

*Calculated by estimating wasting prevalence (<-2 standard deviations below the mean) for a normal Z-curve (mean=0, S.D.=1) compared with a distribution left-shifted by the impact of one diarrhea episode. Note that Troeger et al estimate nearly 3 times the impact per diarrhea episode on wasting compared with stunting, however compared to the Checkley et al study it is not clear whether the impact on weight-for-height is among the wasted vs non-wasted children.

L.8 Impact of past stunting on stunting

Table 10: Odds ratios for continued stunting (<-2 HAZ-score) if stunted in a prior age band

Age in months	Odds Ratio
1-5	45
6-11	361.6
12-23	174.7
24-59	174.7

Source: Cousens et al BMC Public Health 2017 (13).

Appendix B. Regions, countries and interventions

Table 11: Country groupings and packages of interventions considered in this analysis.

Region	Subgroup	Countries	Interventions
Africa West (AFW)	Low income	Burkina Faso, Central African Republic, Chad, The Gambia, Guinea-Bissau, Liberia, Mali, Niger, Sierra Leone, Togo	Small quantity lipid-based nutrition supplements, Cash Transfers, Zinc supplementation, Multiple micronutrient supplementation, IPTp, IYCN, Vitamin A supplementation, Calcium supplementation, Treatment of SAM, Zinc for treatment + ORS, Kangaroo mother care, Delayed cord clamping, IFA staple food fortification, Micronutrient powders, Iron and iodine fortification of salt
	Lower middle income	Benin, Cameroon, Republic of Congo, Côte D'Ivoire, Ghana, Guinea, Mauritania, Nigeria, Senegal	
	Upper middle income	Equatorial Guinea, Gabon	
Eastern and Southern Africa (AFE)	Low income	Burundi, Democratic Republic of Congo, Eritrea, Ethiopia, Madagascar, Malawi, Mozambique, Rwanda, Somalia, South Sudan, Sudan, Uganda	Small quantity lipid-based nutrition supplements, Zinc supplementation, Cash Transfers, Multiple micronutrient supplementation, IPTp, IYCN, Vitamin A supplementation, Calcium supplementation, Treatment of SAM, Zinc for treatment + ORS, Kangaroo mother care, Delayed cord clamping, IFA staple food fortification, Micronutrient powders, Iron and iodine fortification of salt, IFAS for pregnant women
	Lower middle income	Angola, Comoros, Eswatini, Kenya, Lesotho, São Tomé and Príncipe, Tanzania, Zambia, Zimbabwe	
	Upper middle income	Botswana, Mauritius, Namibia, South Africa	
Middle East and North Africa (MENA)	Fragility, conflict and violence, and low-income countries	Djibouti, Iraq, Libya, West Bank & Gaza, Syria, Yemen, Pakistan, Afghanistan	Small quantity lipid-based nutrition supplements, Cash Transfers, Zinc supplementation, Multiple micronutrient supplementation, IPTp, IYCN, Vitamin A supplementation, Calcium supplementation, Treatment of SAM, Zinc for treatment + ORS, Kangaroo mother care, Delayed cord clamping, IFA staple food fortification, Micronutrient powders, Iron and iodine fortification of salt
	Middle income countries	Algeria, Egypt, Iran, Jordan, Lebanon, Morocco, Tunisia	Cash Transfers, Multiple micronutrient supplementation, Zinc supplementation, IYCN, Vitamin A supplementation, Calcium supplementation, Treatment of SAM, Zinc for treatment + ORS, Kangaroo mother care, Delayed cord clamping, IFA staple food

			fortification, Micronutrient powders, Iron and iodine fortification of salt
East Asia and Pacific (EAP)	Lower middle income	Cambodia, Kiribati, Lao People's Democratic Republic, Myanmar, Papua New Guinea, Philippines, Samoa, Solomon Islands, Timor-Leste, Vanuatu, Viet Nam	Small quantity lipid-based nutrition supplements, Cash Transfers, Zinc supplementation, Multiple micronutrient supplementation, IPTp, IYCN, Vitamin A supplementation, Calcium supplementation, Treatment of SAM, Zinc for treatment + ORS, Kangaroo mother care, Delayed cord clamping, IFA staple food fortification, Micronutrient powders, Iron and iodine fortification of salt
	Upper middle income	China, Fiji, Indonesia, Malaysia, Marshall Islands, Mongolia, Thailand, Tonga	
Latin America and Caribbean (LAC)	Lower middle income	Bolivia, Haiti, Honduras, Nicaragua	Cash Transfers, Zinc supplementation, Multiple micronutrient supplementation, IYCN, Vitamin A supplementation, Calcium supplementation, Treatment of SAM, Zinc for treatment + ORS, Kangaroo mother care, Delayed cord clamping, IFA staple food fortification, Micronutrient powders, Iron and iodine fortification of salt
	Upper middle income	Belize, Brazil, Colombia, Costa Rica<br>Cuba, Dominican Republic, Ecuador<br>El Salvador, Grenada, Guatemala<br>Jamaica, Mexico, Paraguay, Peru, Saint Lucia, Saint Vincent and the Grenadines, Suriname	
Eastern Europe and Central Asia (EECA)	Lower middle income	Tajikistan, Ukraine, Uzbekistan, Kyrgyz Republic (Kyrgyzstan)	Cash Transfers, Zinc supplementation, Multiple micronutrient supplementation, IYCN, Vitamin A supplementation, Calcium supplementation, Treatment of SAM, Zinc for treatment + ORS, Kangaroo mother care, Delayed cord clamping, IFA staple food fortification, Micronutrient powders, Iron and iodine fortification of salt
	Upper middle income	Albania, Armenia, Azerbaijan,<br>Bosnia and Herzegovina, Georgia,<br>Kazakhstan, Moldova, Montenegro<br>Serbia, Türkiye (Turkey), Belarus<br>Turkmenistan, North Macedonia (Republic)	
South Asia (SA)	Lower middle income	Bangladesh, Bhutan, India, Nepal, Sri Lanka	Small quantity lipid-based nutrition supplements, Cash Transfers, Zinc supplementation, Multiple micronutrient supplementation, IPTp, IYCN, Vitamin A supplementation, Calcium supplementation, Treatment of SAM, Zinc for treatment + ORS, Kangaroo mother care, Delayed cord clamping, IFA staple food fortification, Micronutrient powders, Iron and iodine fortification of salt
	Upper middle income	Maldives	

Appendix C. Intervention effect sizes

Table 12: Intervention target populations and effect sizes.

Intervention	Target population	Effects	March 2024 new values	March 2024 sources
	CHILDREN			
Cash transfers (conditional)	Children below the poverty line	Reduces stunting 12-59 months	OR 0.808 (0.395-0.956)	Field et al 2024 (14) (CT+BCC). Reduction in stunting (baseline 34% prevalence) of 4.6 percentage points (S.D.=2.1 pc pts) for cash transfer + SBCC.
Delayed umbilical cord clamping	Pregnant women (at birth, but impact is for children <1 month)	Reduces anemia 0-5 months	RR 0.92 (0.87-0.99)	Zhao et al 2019 (15), ≥6 months. While this estimate doesn't correspond to the age bracket there is no recent estimates, and the assumption is that the effect would be at least the same as for ≥6 months
		Reduces anemia 6-12 months	RR 0.92 (0.87-0.99)	Zhao et al 2019 (15), ≥6 months
Infant and young child feeding (IYCF) education/counseling	For children < 1 months	Increases exclusive breastfeeding (home or community settings)	OR 2.17 (1.84-2.56)	Sinha et al 2017 (16)
	For children < 6 months	Increases exclusive breastfeeding (home or community settings)	OR 2.48 (1.99-3.09)	Sinha et al 2017 (16)
	For children 6-23 months	Increases age-appropriate partial breastfeeding (combined delivery: home or community settings and health systems and services)	OR 1.82 (1.36-2.45)	Sinha et al 2017 (16)
Kangaroo mother care	1-5 months	Increases exclusive breastfeeding	OR = 1.39 (1.11-1.74)	Boundy et al. 2016 (17), for exclusive breastfeeding in children 1-6 months
	< 1 months	Reduces neonatal mortality due to prematurity	RR = 0.68 (0.53-0.86)	Sivanandan & Sankar 2023 (18),
Micronutrient powders (i.e. iron sprinkles)	Children 6-59 months, not already receiving LNS	Reduces anemia	RR = 0.69 (0.62-0.77)	Moorthy et al 2020 (19)
Oral rehydration solution (ORS) + Zinc	Children 0-59 months (different quantity by age)	Reduces diarrhea mortality	RRR = 0.24 (0.15-0.38)	Calculated as RRR = 0.31 (0.20-0.49) for ORS (Munos, et al. 2010 (20)), with additional RRR of 0.77 due to the addition of zinc (Walker & Black 2010 (21))
Small quantity lipid-based nutrition supplements (SQ-LNS)	Children 6-23 months old who live in households below the poverty line	Reduces stunting	PR = 0.88 (0.85-0.91)	Dewey et al 2021 (22)
		Reduces the incidence of SAM	PR = 0.69 (0.55-0.86)	Dewey et al 2022 (23), severe wasting
		Reduces the incidence of MAM	PR = 0.86 (0.80-0.93)	Dewey et al 2021 (22), wasting
		Reduces iron deficiency anemia	PR = 0.36 (0.30-0.44)	Wessells et al 2021 (24), iron deficiency anemia
Treatment of severe acute malnutrition (SAM)	Children experiencing SAM	Increases recovery from episode	RR = 1/1.33 (1/1.54, 1/1.16)	Schoonees et al 2019 (25), recovery, RR 1.33 (1.16-1.54)
Vitamin A supplementation	Children 6-59 months	Reduces diarrhea incidence	RR = 0.85 (0.82-0.87)	Imdad et al 2022 (26)

		Reduces diarrhea mortality	RR = 0.88 (0.79-0.98)	Imdad et al 2022 (26)
Zinc supplementation (prophylactic)	Children 1-59 months	Reduces diarrhea incidence	RR 0.91 (0.90-0.93)	Imdad et al 2023 (27)
	PREGNANT WOMEN			
Calcium supplementation	Pregnant women	Reduces pre-term births	RR = 0.76 (0.60-0.97)	Hofmeyr et al 2023 (28)
Iron and folic acid supplementation	Pregnant women	Reduces anemia	RR = 0.51 (0.38-0.70)	Hansen et al, 2023 (29), for anemia in pregnant women
		Reduces SGA birth outcomes	RR = 0.39 (0.17-0.86)	Hansen et al, 2023 (29)
Intermittent preventative treatment of malaria during pregnancy	Pregnant women in areas where there is malaria risk	Reduces anemia<br>Reduces SGA birth outcomes	RR = 0.90 (0.84-0.95)<br>RR = 0.65 (0.55-0.77)	Moorthy et al 2020 (19)<br>RRR = 0.65 (0.55-0.77) for SGA birth outcomes (Eisele et al. 2010 (30))
Multiple micronutrient supplementation	Pregnant women	Reduces anemia	RR = 0.51 (0.38-0.70)	Hansen et al, 2023 (29), evidence suggests not statistically significantly different from iron and folic acid supplementation
		Reduces risk of SGA birth outcomes	RR = 0.90 (0.84-0.96) vs IFAS	Hofmeyr et al 2023 (28), for MMS vs IFAS
		Reduces risk of stillbirths	RR = 0.91 (0.86-0.98)	Hofmeyr et al 2023 (28)
	GENERAL			
Iron and folic acid fortification (wheat, maize or rice)	Everyone (except children < 6 moths)	Reduces anemia	OR = 0.976 (0.975-0.978)	Barkley et al. 2015 (31)
		Reduces neonatal mortality	RR = 0.87 (0.84-0.89)	Blencowe et al. 2010 (32), neonatal mortality, prevention of neural tube defects
Iron and iodine fortification of salt	Everyone (except children < 6 moths)	Reduces anemia	RR 0.79 (0.66-0.94)	Baxter et al 2022 (33)
		Reduces neonatal mortality	OR = 0.976 (0.975-0.978)	Barkley et al. 2015 (31)

Appendix D. Intervention unit costs

Unit cost data for interventions were sourced from scientific literature, grey literature, World Bank reports and internal documents. These unit costs were available for some countries across most regions and were extrapolated to other countries within the same region using the following assumptions:

1. **Country-Specific Estimates**: If a country had unit cost estimates for any of the interventions included in the package, these estimates were used for calculations.
2. **Regional Estimates**: If a country lacked unit cost estimates for a specific intervention but estimates were available for other countries in the region, two pathways were considered:
   - Pathway A: For interventions with estimates from three or more countries, excluding outliers, a regional average was calculated and applied to countries without national unit costs.
   - Pathway B: For interventions with estimates from two or fewer countries, excluding outliers, an average of the regional mean and the global median from the Investment Framework for Nutrition 2024 was calculated and applied to countries without national unit costs.
3. **Global Estimates**: For interventions where no country in the region had available unit costs, the global median from the Investment Framework for Nutrition 2024 was applied.

All costs were inflated from their base year to 2023 US dollars.

Table 13: Proportion of country cost values considered for regional estimates

	**Africa West**	**Eastern and Southern Africa**	**Latin America and Caribbean**	**East Asia and Pacific**	**Middle East and North Africa**	**Eastern Europe and Central Asia**	**South Asia**
	% of countries with cost values and n (excludes outliers)						
Calcium supplementation	10%, n=3*	0%, n=0	0%, n=0	10%, n=2	0%, n=0	6%, n=1	17%, n=1
Cash transfers	29%, n=6*	31%, n=8	5%, n=1	10%, n=2	13%, n=2	6%, n=1	17%, n=1
Delayed umbilical cord clamping	29%, n=6	4%, n=1	0%, n=0	5%, n=1	0%, n=0	0%, n=0	0%, n=0
Iron and folic acid fortification (wheat, maize or rice)	38%, n=8	12%, n=3	9%, n=2	10%, n=2	0%, n=0	6%, n=1	17%, n=1
Iron and iodine fortification of salt	10%, n=2	8%, n=2	0%, n=0	5%, n=1	13%, n=2	0%, n=0	33%, n=2
Intermittent preventative treatment of malaria during pregnancy	24%, n=5	12%, n=3	NA	5%, n=1	0%, n=0	NA	0%, n=0
Infant and young child feeding (IYCF) education/counseling	29%, n=6*	31%, n=8	5%, n=1	10%, n=2	13%, n=2	6%, n=1	17%, n=1

Kangaroo mother care	29%, n=6	8%, n=2	0%, n=0	5%, n=1	0%, n=0	0%, n=0	0%, n=0
Micronutrient powders (i.e. iron sprinkles)	19%, n=4*	27%, n=7	5%, n=1	5%, n=1	7%, n=1	6%, n=1	17%, n=1
Multiple micronutrient supplementation	19%, n=4	4%, n=1	9%, n=2	10%, n=2	7%, n=1	6%, n=1	17%, n=1
Small quantity lipid-based nutrition supplements (SQ-LNS)	29%, n=6*	12%, n=3	NA	0%, n=0	0%, n=0	NA	0%, n=0
Treatment of SAM	33%, n=7	27%, n=7	5%, n=1	5%, n=1	7%, n=1	6%, n=1	33%, n=2
Vitamin A supplementation	33%, n=7	31%, n=8	9%, n=2	10%, n=2	13%, n=2	6%, n=1	33%, n=2
Zinc supplementation	10%, n=2	8%, n=2	0%, n=0	5%, n=1	0%, n=0	0%, n=0	0%, n=0
Oral rehydration solution (ORS) + Zinc	29%, n=6	31%, n=8	0%, n=0	5%, n=1	7%, n=1	6%, n=1	33%, n=2
Iron and folic acid supplementation	NA	27%, n=7	NA	NA	NA	NA	NA

Note: the following number of countries were considered in each region for proportion estimations: Africa West n=21, Eastern and Southern Africa n=26, Latin America and Caribbean n=22, East Asia and Pacific n=21, Middle East and North Africa n=15, Eastern Europe and Central Asia n=18. South Asia, n=6.

* Denotes that 1 outlier was found and subtracted before proportion estimation. Outliers were identified through outliers include visual inspection via box plots.

Appendix E. Intervention coverages

Baseline coverage of interventions was primarily derived from the most recent demographic and health surveys (DHS), covering indicators such as IPTp, micronutrient powders for children, vitamin A supplementation, zinc plus oral rehydration solution (ORS) for treatment of diarrhea and treatment of SAM.

While DHS is a key data source, it does not provide coverage data for all interventions. In such cases proxies for intervention coverage were used. For example, coverage for infant and young child nutrition (IYCN) counselling is not directly available from the DHS, but it was assumed that a significant part of IYCN counselling occurs during vaccination visits, and therefore a child who received all age-appropriate vaccinations would have been provided opportunities for a caregiver to receive nutrition counselling during the vaccination service. The indicator for the percentage of children 12-35 months old who had received all age-appropriate vaccinations were therefore assumed as the coverage for IYCN counselling.

In cases where DHS data were not available for recent coverage estimates, default values from the Lives Saved Tool (LiST) were used. For some interventions, LiST was used as the primary source of information, including iron and iodine fortification of salt, ORS coverage, delayed cord clamping, treatment of SAM.

Reliable data on the coverage of staple food fortification (e.g. rice, wheat flour, maize flour) is limited. Data on wheat flour fortification was used as a proxy for coverage across all regions, based on the assumption that wheat is the most widely consumed staple globally. The most recent FAO Supply Utilization Accounts were used to identify wheat fortification coverage in each country. If wheat fortification was mandatory, baseline coverage was set at 50%; if fortification was voluntary, coverage was set at 25%. If there is no or unknown fortification standard, we assume there to be minimal consumption of the fortified food and use baseline coverage of zero for the analysis.

Coverage of new interventions not yet widely scaled, such as calcium supplementation and multiple micronutrient supplementation for pregnant women, SQ-LNS for children, kangaroo mother care, and prophylactic zinc supplementation for children, was assumed to be zero.

For the scale-up of interventions, coverage was linearly interpolated from baseline to a maximum of 90% of the intervention target population over the five-year period 2025-2029. This assumes that 90% coverage can be achieved using routine delivery approaches, while the remaining 10% may be harder to reach due to issues of accessibility and acceptability of the interventions, and that it would require significantly more than the average marginal cost for the routine delivery of the interventions.

Appendix F. Economic benefit calculations

Stunting

The economic benefits of reduced stunting were estimated as increased future workforce productivity from stunting cases averted (assuming a 66% lower lifetime income per capita, based on Hoddinott et al. (34)) and child deaths. Average income was based on country-specific GDP per capita, assuming 90% of lifetime earnings are realized (34), and adjusting for country- and age-specific all-cause mortality(35) among the children whose deaths/stunting were averted (since not all would survive their entire working lives). Country GDP was assumed to grow at 3% per annum, working age was assumed to be 18-65 years, and 3% per annum discounting of costs and benefits was applied.

Wasting

The economic benefits of reduced wasting were estimated as increased future workforce productivity from child deaths averted. Average income was based on country-specific GDP per capita, assuming 90% of lifetime earnings are realized (34), and adjusting for country- and age-specific all-cause mortality (35) among the children whose deaths were averted (since not all would survive their entire working lives). Country GDP was assumed to grow at 3% per annum, working age was assumed to be 18-65 years, and 3% per annum discounting of costs and benefits was applied.

Child Anemia

The economic benefits of reduced child anemia were estimated as increased future workforce productivity from child anemia cases averted (assuming a 2.5% lower lifetime income per capita, based on Horton and Ross 2003 (36)) and child deaths averted. Average income was based on country-specific GDP per capita, assuming 90% of lifetime earnings are realized (34), and adjusting for country- and age-specific all-cause mortality (35) among the children whose anemia/deaths were averted (since not all would survive their entire working lives). Country GDP was assumed to grow at 3% per annum, working age was assumed to be 18-65 years, and 3% per annum discounting of costs and benefits was applied.

Maternal Anemia

The economic benefits of reduced maternal anemia were estimated as increased workforce productivity from maternal anemia cases averted, as well as both maternal deaths and child deaths averted. For maternal anemia averted, a 5% gain for light labor and a 12% gain for heavy labor (36) was assumed, with average wage based on GDP per capita, assuming 90% lifetime earnings are realized (34), and adjusted for country-specific female labor force participation (35); productivity gains were only applied to country-specific wage share of GDP for manual occupations (37), from which a 60% share for light labor and 8% for heavy labor was assumed(36), and for the first half of pregnancy. For maternal deaths averted, average wage was based on GDP per capita, assuming 90% lifetime earnings are realized and adjusting for female labor force participation. Country- and age-specific all-cause mortality was applied to those women whose deaths were averted (since not all would survive the entire remainder of working lives), using country-specific distributions of age at pregnancy. For child deaths averted, economic benefits were based on future earnings, with average wage based on GDP per capita, assuming 90% lifetime earnings are realized, and similarly adjusting for country- and age-

specific all-cause mortality. Country GDP was assumed to grow at 3% per annum, working age was assumed to be 18-65 years, and 3% per annum discounting of costs and benefits was applied.

Breastfeeding

The economic benefits of increased breastfeeding were estimated as increased future workforce productivity from children who were exclusively breastfed (assuming a 0.8 point increase in IQ due to exclusive breastfeeding (38) and a 1.07% increase in lifetime earnings for each additional percentage point increase (39)) and child deaths averted. Average income was based on country-specific GDP per capita, assuming 90% of lifetime earnings are realized (34), and adjusting for country- and age-specific all-cause mortality (35) among the children who were exclusively breastfed/whose deaths were averted (since not all would survive their entire working lives). Country GDP was assumed to grow at 3% per annum, working age was assumed to be 18-65 years, and 3% per annum discounting of costs and benefits was applied.

Small for gestational age births

The economic benefits of low birthweight reductions were estimated as increased future workforce productivity from averted low birthweight births (assuming a 7.5% increase in future workforce productivity (40)) and child deaths averted. Average income was based on country-specific GDP per capita, assuming 90% of lifetime earnings are realized (34), and adjusting for country- and age-specific all-cause mortality (35) among the children whose low birthweight/deaths were averted (since not all would survive their entire working lives). Country GDP was assumed to grow at 3% per annum, working age was assumed to be 18-65 years, and 3% per annum discounting of costs and benefits was applied.

Appendix G. Additional regional outputs

Table 14: Baseline and additional spending by region and intervention, total over 2025-2034. Costs are in 2023 US$ and include 3% per annum discounting.

	Africa West		Eastern and Southern Africa		Middle East and North Africa		East Asia and Pacific		South Asia		Latin America and Caribbean		Eastern Europe and Central Asia	
	Baseline (million US$)	Additional (million US$)	Baseline (million US$)	Additional (million US$)	Baseline (million US$)	Additional (million US$)	Baseline (million US$)	Additional (million US$)	Baseline (million US$)	Additional (million US$)	Baseline (million US$)	Additional (million US$)	Baseline (million US$)	Additional (million US$)
Small quantity lipid-based nutrition supplements	$0	$2,750	$0	$5,793	$0	$1,453	$0	$575	$0	$2,653	$0	$0	$0	$0
Cash transfers	$0	$338	$0	$680	$0	$233	$0	$10	$0	$408	$0	$228	$0	$81
Zinc supplementation	$0	$964	$0	$4,180	$0	$1,553	$0	$1,734	$0	$2,504	$0	$734	$0	$334
Multiple micronutrient supplementation	$0	$1,271	$0	$1,637	$0	$2,433	$0	$2,327	$0	$4,032	$0	$443	$0	$276
IPTp	$193	$79	$134	$85	$2	$36	$1	$8	$13	$72	$0	$0	$0	$0
IYCF 1	$461	$721	$979	$1,503	$734	$645	$758	$1,487	$1,965	$1,114	$821	$1,684	$232	$204
Vitamin A supplementation	$173	$45	$367	$137	$90	$96	$258	$271	$107	$21	$25	$323	$60	$66
Calcium supplementation	$0	$874	$0	$1,644	$0	$570	$0	$242	$0	$575	$0	$128	$0	$83
Treatment of SAM	$935	$2,728	$637	$4,315	$99	$6,234	$14	$4,912	$6	$18,329	$5	$282	$0	$517
Zinc for treatment + ORS	$31	$84	$26	$96	$12	$124	$15	$116	$9	$20	$2	$36	$1	$13
Kangaroo mother care	$0	$18	$0	$102	$0	$67	$0	$92	$0	$103	$0	$29	$0	$13
Delayed cord clamping	$0	$7	$0	$29	$0	$4	$0	$23	$0	$7	$0	$2	$0	$1
IFA fortification of wheat flour	$152	$107	$194	$293	$157	$549	$468	$768	$142	$263	$225	$141	$53	$150
Micronutrient powders	$878	$1,519	$126	$2,732	$70	$1,192	$494	$4,603	$28	$3,540	$227	$827	$18	$500
Iron and iodine fortification of salt	$177	$711	$255	$1,204	$31	$67	$44	$459	$1	$213	$60	$111	$18	$54
IFAS for pregnant women	$0	$0	$0	$0	$0	$0	$0	$0	$0	$0	$0	$0	$0	$0

Table 15: Baseline health and nutrition outcomes by region, for 2025-2034. Aggregated over included countries within each region.

Region	Africa West	Eastern and Southern Africa	Middle East and North Africa	East Asia and Pacific	South Asia	Latin America and Caribbean	Eastern Europe and Central Asia
Child deaths	20,037,000	17,627,000	9,398,000	3,918,000	9,949,000	1,542,000	570,000
Stunting cases	54,753,000	89,947,000	55,039,000	73,671,000	96,143,000	10,333,000	3,564,000
Wasting episodes	27,109,000	33,799,000	27,445,000	38,324,000	96,443,000	2,654,000	2,157,000
Child anemia cases	56,017,000	79,093,000	47,541,000	25,876,000	82,915,000	14,651,000	7,692,000
Small for gestational age births	1,264,000	2,360,000	3,499,000	1,384,000	9,046,000	513,000	233,000
Children exclusively breastfed	48,818,000	113,622,000	55,060,000	80,606,000	136,525,000	23,146,000	9,719,000
Maternal anemia cases	65,316,000	74,807,000	56,971,000	69,250,000	92,695,000	19,443,000	9,755,000
Maternal deaths	1,151,000	796,000	261,000	168,000	259,000	58,000	8,000

Appendix H. Appendix references

1. Katz J, Lee AC, Kozuki N, Lawn JE, Cousens S, Blencowe H, et al. Mortality risk in preterm and small-for-gestational-age infants in low-income and middle-income countries: a pooled country analysis. Lancet (London, England). 2013;382(9890):417-25.
2. Olofin I, McDonald CM, Ezzati M, Flaxman S, Black RE, Fawzi WW, et al. Associations of suboptimal growth with all-cause and cause-specific mortality in children under five years: a pooled analysis of ten prospective studies. PloS one. 2013;8(5):e64636.
3. Timing of initiation, patterns of breastfeeding, and infant survival: prospective analysis of pooled data from three randomised trials. The Lancet Global health. 2016;4(4):e266-75.
4. Lamberti LM, Fischer Walker CL, Noiman A, Victora C, Black RE. Breastfeeding and the risk for diarrhea morbidity and mortality. BMC public health. 2011;11 Suppl 3(Suppl 3):S15.
5. Lamberti LM, Zakarija-Grković I, Fischer Walker CL, Theodoratou E, Nair H, Campbell H, et al. Breastfeeding for reducing the risk of pneumonia morbidity and mortality in children under two: a systematic literature review and meta-analysis. BMC public health. 2013;13 Suppl 3(Suppl 3):S18.
6. Heidkamp R, Guida R, Phillips E, Clermont AJTJon. The Lives Saved Tool (LiST) as a model for prevention of anemia in women of reproductive age. 2017;147(11):2156S-62S.
7. LiST. LiST technical note. Accessed 11 June 2025 from https://static1.squarespace.com/static/5bbba6574d8711a7dcafa92a/t/5c93e9b9f4e1fc34bdbe9cc8/1553197497868/Birth+outcomes+on+stunting+at+1+mo.pdf. 2016.
8. Christian P, Lee SE, Donahue Angel M, Adair LS, Arifeen SE, Ashorn P, et al. Risk of childhood undernutrition related to small-for-gestational age and preterm birth in low- and middle-income countries. Int J Epidemiol. 2013;42(5):1340-55.
9. Kozuki N, Lee AC, Katz J. Moderate to severe, but not mild, maternal anemia is associated with increased risk of small-for-gestational-age outcomes. J Nutr. 2012;142(2):358-62.
10. Xiong X, Buekens P, Alexander S, Demianczuk N, Wollast E. Anemia during pregnancy and birth outcome: a meta-analysis. American journal of perinatology. 2000;17(3):137-46.
11. Checkley W, Buckley G, Gilman RH, Assis AM, Guerrant RL, Morris SS, et al. Multi-country analysis of the effects of diarrhoea on childhood stunting. Int J Epidemiol. 2008;37(4):816-30.
12. Troeger C, Colombara DV, Rao PC, Khalil IA, Brown A, Brewer TG, et al. Global disability-adjusted life-year estimates of long-term health burden and undernutrition attributable to diarrhoeal diseases in children younger than 5 years. The Lancet Global health. 2018;6(3):e255-e69.
13. Cousens S, Perin J, Christian P, Wu LS, Soofi S, Bhutta Z, et al. Modelling stunting in LiST: the effect of applying smoothing to linear growth data. BMC public health. 2017;17(Suppl 4):778.
14. Field E. M., Maffioli E. M. Are Behavioral Change Interventions Needed to Make Cash Transfer Programs Work for Children? Experimental Evidence from Myanmar. In: National Bureau of Economic Research, editor. Cambridge, MA2021.
15. Zhao Y, Hou R, Zhu X, Ren L, Lu H. Effects of delayed cord clamping on infants after neonatal period: A systematic review and meta-analysis. Int J Nurs Stud. 2019;92:97-108.
16. Sinha B., Chowdhury R., Upadhyay R. P., Taneja S., Martines J., Bahl R., et al. Integrated Interventions Delivered in Health Systems, Home, and Community Have the Highest Impact on Breastfeeding Outcomes in Low- and Middle-Income Countries. The Journal of Nutrition. 2017;147(11):2179S-87S.
17. Boundy EO, Dastjerdi R, Spiegelman D, Fawzi WW, Missmer SA, Lieberman E, et al. Kangaroo Mother Care and Neonatal Outcomes: A Meta-analysis. Pediatrics. 2016;137(1).
18. Sivanandan S., Sankar M. J. Kangaroo mother care for preterm or low birth weight infants: a systematic review and meta-analysis. BMJ Global Health. 2023;8:1-13.
19. Moorthy D, Merrill R, Namaste S, Iannotti L. The Impact of Nutrition-Specific and Nutrition-Sensitive Interventions on Hemoglobin Concentrations and Anemia: A Meta-review of Systematic Reviews. Advances in nutrition (Bethesda, Md). 2020;11(6):1631-45.

20. Munos MK, Walker CL, Black RE. The effect of oral rehydration solution and recommended home fluids on diarrhoea mortality. Int J Epidemiol. 2010;39 Suppl 1(Suppl 1):i75-87.
21. Walker C. L. F., Black RE. Zinc for the treatment of diarrhoea: effect on diarrhoea morbidity, mortality and incidence of future episodes. International Journal of Epidemiology. 2010;39:i63–i9.
22. Dewey KG, Stewart CP, Wessells KR, Prado EL, Arnold CD. Small-quantity lipid-based nutrient supplements for the prevention of child malnutrition and promotion of healthy development: overview of individual participant data meta-analysis and programmatic implications. Am J Clin Nutr. 2021;114(Suppl 1):3s-14s.
23. Dewey KG, Arnold CD, Wessells KR, Prado EL, Abbeddou S, Adu-Afarwuah S, et al. Preventive small-quantity lipid-based nutrient supplements reduce severe wasting and severe stunting among young children: an individual participant data meta-analysis of randomized controlled trials. Am J Clin Nutr. 2022;116(5):1314-33.
24. Wessells KR, Arnold CD, Stewart CP, Prado EL, Abbeddou S, Adu-Afarwuah S, et al. Characteristics that modify the effect of small-quantity lipid-based nutrient supplementation on child anemia and micronutrient status: an individual participant data meta-analysis of randomized controlled trials. Am J Clin Nutr. 2021;114(Suppl 1):68s-94s.
25. Schoonees A, Lombard MJ, Musekiwa A, Nel E, Volmink J. Ready-to-use therapeutic food (RUTF) for home-based nutritional rehabilitation of severe acute malnutrition in children from six months to five years of age. Cochrane Database Syst Rev. 2019;5(5):Cd009000.
26. Imdad A, Mayo-Wilson E, Haykal MR, Regan A, Sidhu J, Smith A, et al. Vitamin A supplementation for preventing morbidity and mortality in children from six months to five years of age. Cochrane Database Syst Rev. 2022;3(3):Cd008524.
27. Imdad A, Rogner J, Sherwani RN, Sidhu J, Regan A, Haykal MR, et al. Zinc supplementation for preventing mortality, morbidity, and growth failure in children aged 6 months to 12 years. Cochrane Database Syst Rev. 2023;3(3):Cd009384.
28. Hofmeyr GJ, Lawrie TA, Atallah Á N, Torloni MR. Calcium supplementation during pregnancy for preventing hypertensive disorders and related problems. Cochrane Database Syst Rev. 2018;10(10):Cd001059.
29. Hansen R, Sejer EPF, Holm C, Schroll JB. Iron supplements in pregnant women with normal iron status: A systematic review and meta-analysis. Acta Obstet Gynecol Scand. 2023;102(9):1147-58.
30. Eisele TP, Larsen D, Steketee RW. Protective efficacy of interventions for preventing malaria mortality in children in Plasmodium falciparum endemic areas. Int J Epidemiol. 2010;39 Suppl 1(Suppl 1):i88-101.
31. Barkley JS, Wheeler KS, Pachón H. Anaemia prevalence may be reduced among countries that fortify flour. The British journal of nutrition. 2015;114(2):265-73.
32. Blencowe H., Cousens S., Modell B., Lawn J. Folic acid to reduce neonatal mortality from neural tube disorders. International Journal of Epidemiology. 2010;39(Suppl 1):i110–21.
33. Baxter JB, Carducci B, Kamali M, Zlotkin SH, Bhutta ZA. Fortification of salt with iron and iodine versus fortification of salt with iodine alone for improving iron and iodine status. Cochrane Database Syst Rev. 2022;4(4):Cd013463.
34. Hoddinott J, Alderman H, Behrman JR, Haddad L, Horton S. The economic rationale for investing in stunting reduction. Maternal & child nutrition. 2013;9 Suppl 2(Suppl 2):69-82.
35. World Bank. World Development Indicators. Accessed 13 May 2025: https://databank.worldbank.org/source/world-development-indicators.
36. Horton S, Ross J. The economics of iron deficiency. Food Policy. 2003;28(1):51-75.
37. International Labour Organization. ILOSTAT. https://ilostat.ilo.org/. 2024.
38. Wigg NR, Tong S, McMichael AJ, Baghurst PA, Vimpani G, Roberts R. Does breastfeeding at six months predict cognitive development? Australian and New Zealand journal of public health. 1998;22(2):232-6.
39. Hanushek EA, Woessmann L. The role of cognitive skills in economic development. Journal of economic literature. 2008;46(3):607-68.

40. Alderman H, Behrman JR. Reducing the incidence of low birth weight in low-income countries has substantial economic benefits. The World Bank Research Observer. 2006;21(1):25-48.
